# The peptide transporter 1a of the zebrafish *Danio rerio*, an emerging model in nutrigenomics and nutrition research: molecular characterization, functional properties, and expression analysis

**DOI:** 10.1186/s12263-019-0657-3

**Published:** 2019-12-19

**Authors:** Francesca Vacca, Amilcare Barca, Ana S. Gomes, Aurora Mazzei, Barbara Piccinni, Raffaella Cinquetti, Gianmarco Del Vecchio, Alessandro Romano, Ivar Rønnestad, Elena Bossi, Tiziano Verri

**Affiliations:** 1 Department of Biotechnology and Life Sciences, University of Insubria, via J.H. Dunant 3, 21100 Varese, Italy; 2 Department of Biological and Environmental Sciences and Technologies, University of Salento, via Provinciale Lecce-Monteroni, I-73100 Lecce, Italy; 3 Department of Biological Sciences, University of Bergen, P.O. Box 7803, NO-5020 Bergen, Norway; 4 Present address: Physiopathology of Reproduction and IVF Unit, Nardò Hospital, Nardò Health and Social Care District, Lecce Local Health Agency, I-73048 Nardò, Lecce Italy; 5 Division of Neuroscience, Institute of Experimental Neurology, IRCCS San Raffaele Scientific Institute, I-20132 Milan, Italy

**Keywords:** Di/tripeptide transport(ers), Dietary protein, Electrogenic transport, Heterologous expression, Peptide absorption, pH-dependence, Teleost fish, Whole genome duplication, *Xenopus laevis* oocytes

## Abstract

**Background:**

Peptide transporter 1 (PepT1, *alias* Slc15a1) mediates the uptake of dietary di/tripeptides in all vertebrates. However, in teleost fish, more than one PepT1-type transporter might function, due to specific whole genome duplication event(s) that occurred during their evolution leading to a more complex paralogue gene repertoire than in higher vertebrates (tetrapods).

**Results:**

Here, we describe a novel di/tripeptide transporter in the zebrafish (*Danio rerio*), i.e., the zebrafish peptide transporter 1a (PepT1a; also known as Solute carrier family 15 member a1, Slc15a1a), which is a paralogue (78% similarity, 62% identity at the amino acid level) of the previously described zebrafish peptide transporter 1b (PepT1b, *alias* PepT1; also known as Solute carrier family 15 member 1b, Slc15a1b). Also, we report a basic analysis of the *pept1a* (*slc15a1a*) mRNA expression levels in zebrafish adult tissues/organs and embryonic/early larval developmental stages. As assessed by expression in *Xenopus laevis* oocytes and two-electrode voltage clamp measurements, zebrafish PepT1a, as PepT1b, is electrogenic, Na^+^-independent, and pH-dependent and functions as a low-affinity system, with *K*_0.5_ values for Gly-Gln at − 60 mV of 6.92 mmol/L at pH 7.6 and 0.24 mmol/L at pH 6.5 and at − 120 mV of 3.61 mmol/L at pH 7.6 and 0.45 mmol/L at pH 6.5. Zebrafish *pept1a* mRNA is highly expressed in the intestine and ovary of the adult fish, while its expression in early development undergoes a complex trend over time, with *pept1a* mRNA being detected 1 and 2 days post-fertilization (dpf), possibly due to its occurrence in the RNA maternal pool, decreasing at 3 dpf (~ 0.5-fold) and increasing above the 1–2 dpf levels at 4 to 7 dpf, with a peak (~ 7-fold) at 6 dpf.

**Conclusions:**

We show that the zebrafish PepT1a-type transporter is functional and co-expressed with *pept1b* (*slc15a1b*) in the adult fish intestine. Its expression is also confirmed during the early phases of development when the yolk syncytial layer is present and yolk protein resorption processes are active. While completing the missing information on PepT1-type transporters function in the zebrafish, these results open to future investigations on the similar/differential role(s) of PepT1a/PepT1b in zebrafish and teleost fish physiology.

## Background

The intestinal peptide transporter 1 (PepT1) plays a major role in protein nutrition as it mediates the luminal-to-cellular uptake of dietary amino acids in a di- and tripeptide (di/tripeptide) form at the brush-border membrane of the enterocytes [1, 2]. In this way, PepT1 allows absorption of large fractions of exogenous ingested proteins of animal, plant, and microorganism origin and/or endogenous luminal resident proteins of epithelial and microorganism origin, as they are terminally degraded by digestive and/or microbial enzymes [3–5]. PepT1 is also responsible for the absorption of orally active peptidomimetics, including β-lactam antibiotics and selected pro-drugs [2, 6, 7].

PepT1 belongs to the peptide transporter family [8], which members are found from bacteria to vertebrates [8–11]. In humans, it is referred to as the Solute Carrier 15 (SLC15) family member A1 (SLC15A1) [1, 2]. In higher vertebrates, PepT1 is a Na^+^-independent, H^+^-dependent electrogenic symporter, and by coupling substrate uptake to H^+^-movement down an inwardly directed electrochemical H^+^-gradient, it allows transport of peptides across the plasma membrane even against a substrate concentration gradient. The transport responds to membrane potential and extracellular pH, with extracellular pH-optima varying between 4.5 and 6.5 depending on the net charge of the transported substrate [1, 2, 10]. PepT1 function has also been described in detail in teleost fish [12, 13]. Zebrafish (*Danio rerio*) PepT1, the first teleost PepT1-type transporter cloned and functionally characterized [14], exhibited a unique pH dependence, with neutral-to-alkaline extracellular pH increasing its maximal transport rate (for information on human vs. zebrafish PepT1-type transporters and of their major features in the larger context of the human and zebrafish SLC15 transporters see Table 1). However, studies on European sea bass (*Dicentrarchus labrax*) [25], Atlantic salmon (*Salmo salar*) [26], and Antarctic icefish (*Chionodraco hamatus*) [27] PepT1 transporters revealed a more standard behavior with respect to the pH-optimum, with maximal transport rates independent of the extracellular pH in the alkaline to neutral-to-slightly-acidic range [25–28]. With respect to substrate specificity, as in higher vertebrates, all teleost PepT1 transporters also mediated the uptake of neutral and charged di/tripeptides [26, 28, 29].

**Table 1:** The Solute Carrier 15 (proton oligopeptide cotransporter) family members in human (*Homo sapiens*) and zebrafish (*Danio rerio*)

Human							Zebrafish				
From *http://www.bioparadigms.org*						From *http://www.guidetopharmacology.org*	From *http://www.ncbi.nlm.nih.gov/gene*	*https://www.ncbi.nlm.nih.gov/unigene/*	From *http://zfin.org*		
SLC name	Protein name	Aliases	Transport type	Substrates	Tissue and cellular expression	Substrates	slc name	EST profile	Tissue and cellular expression	Stage range	References
SLC15A1	PEPT1	Oligopeptide transporter 1, H^+^-peptide transporter 1	C/H^+^	Di- and tripeptides, protons, β-lactam antibiotics	Small intestine, kidney, pancreas, bile duct, liver	Endogenous substrates: 5-aminolevulinic acid, dipeptides, protons, tripeptides. Other substrates: fMet-Leu-Phe, muramyl dipeptide, D-Ala-Lys-AMCA, β-Ala-Lys-AMCA, His-Leu-lopinavir, alafosfalin.	*slc15a1a (Chr 9)*	–	Details in this study	Details in this study	This study
							*slc15a1b (Chr 6)*	Developmental stage|larval > adult Adult|intestine	Digestive system, gut, intestinal bulb, intestinal bulb enterocyte, intestinal epithelium, liver, muscle, squamous epithelial cell, whole organism	Prim-5 to adult	[14–22]
SLC15A2	PEPT2	Oligopeptide transporter 2, H^+^-peptide transporter 2	C/H^+^	Di- and tripeptides, protons, β-lactam antibiotics	Apical surface of epithelial cells from kidney and choroid plexus; neurons, astrocytes (neonates), lung, mammary gland, spleen, enteric nervous system	Endogenous substrates: 5-aminolevulinic acid, dipeptides, protons, tripeptides. Other substrates: muramyl dipeptide, D-Ala-Lys-AMCA, β-Ala-Lys-AMCA, alafosfalin, γ-iE-DAP.	*slc15a2 (Chr 9)*	Developmental stage|adult Adult|intestine >> kidney > reproductive system	Brain, eye, gill, gut, kidney, musculature system, otic vesicle, semicircular canal, ventricular system, whole organism	26+ somites to day 6; days 30–44; adult	[21, 23]
SLC15A3	PHT2	Peptide/histidine transporter 2, PTR3	C/H^+^	Di- and tripeptides, protons, β-lactam antibiotics	Lung, spleen, thymus, intestine (faintly in brain, liver, adrenal gland, heart)	Endogenous substrates: L-histidine, dipeptides, protons, tripeptides. Other substrates: muramyl dipeptide, MDP-rhodamine, Tri-DAP	n.p.	n.p.	n.p.	n.p.	n.p.
SLC15A4	PHT1	Peptide/histidine transporter 1, PTR4	C/H^+^	Di- and tripeptides, protons, β-lactam antibiotics	Brain, eye, intestine (faintly in lung and spleen)	Endogenous substrates: L-histidine, carnosine Other substrates: valacyclovir, muramyl dipeptide, His-Leu-lopinavir, glycyl-sarcosine, MDP-rhodamine, Tri-DAP, C12-iE-DAP	*slc15a4 (Chr 8)*	Developmental stage|adult Adult|brain ≈ reproductive system ≥ fin	Epidermis, eye, immature eye, midbrain, periderm, ventricular zone, yolk syncytial layer	50%-epiboly to Long-pec	[24]

Abbreviations for transport type: *C* cotransporter, *n.p.* not present in the zebrafish genome

With several teleost genomes available in databanks, it was progressively clear that teleost PepT1-type proteins are the result of a gen(om)e duplication event, and, after an initial description of partial nucleotide sequences [30], the idea that in teleost genomes a *peptide transporter 1a* (*pept1a*; also known as *solute carrier family 15 member 1a*, *slc15a1a*) gene occurs beside a *peptide transporter 1b* (*pept1b*, *alias pept1*; also known as *solute carrier family 15 member 1b*, *slc15a1b*) gene fully emerged. Also, it was clear that all the functional data available in teleosts literature referred to PepT1b-type transporters only [12, 13].

The question whether or not teleost PepT1a-type transporters are functional has been answered recently, with first cloning, analysis of sequence, tissue expression of Atlantic salmon *pept1a* (*slc15a1a*), and its functional characterization in terms of transport kinetics and substrate specificity [31]. In this study, we report information on the functional expression of zebrafish *pept1a* (*slc15a1a*) and compare it to its species paralog *pept1b* (*slc15a1b*). These findings extend the data on the Atlantic salmon *pept1a* (*slc15a1a*) and indicate that this gene expresses a di/tripeptide transporter transporting peptide substrates across the membranes along the intestinal tract epithelial layer in feeding fish. Possibly, it also operates in an extra-embryonic tissue such as the yolk syncytial layer during the pre-feeding stages. Notably, our data fill the missing information and define the functional picture of the whole set of PepT-type (i.e., the PepT1- and PepT2-type; see Table 1) transporters in an “alternative model in nutrigenomics” such as the zebrafish.

## Results

### Sequence analysis

**Fig. 1:**
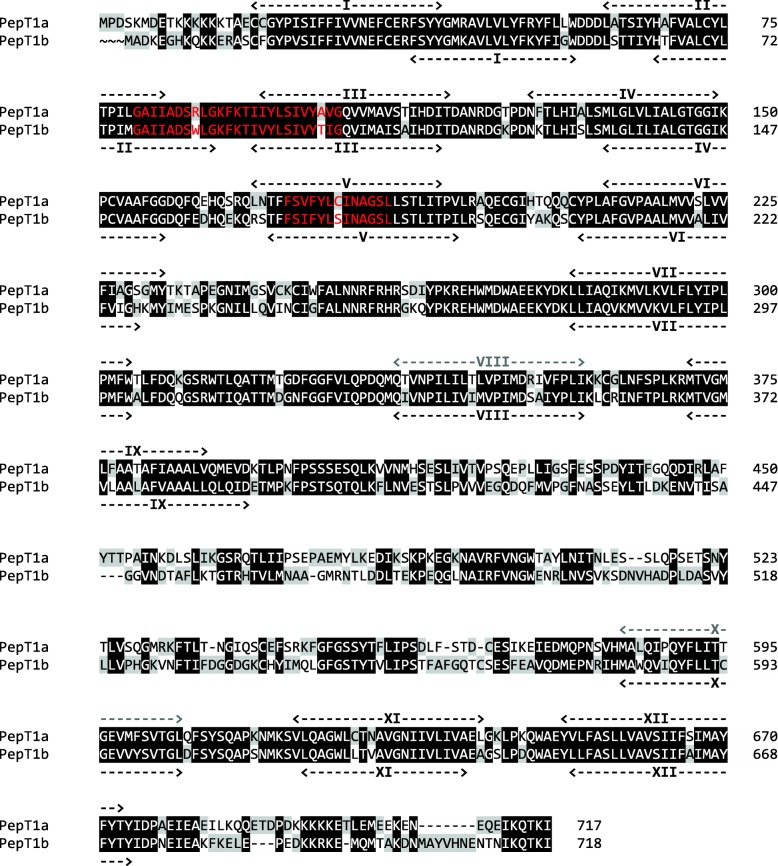
Pairwise alignment between zebrafish PepT1a (Slc15a1a) and PepT1b (Slc15a1b) amino acid sequences obtained by using Clustal Omega and edited using GeneDoc 2.7 software. The predicted conserved PTR2 family proton/oligopeptide symporter signatures (in zebrafish PepT1a, motif 1—PROSITE pattern PS01022—amino acid residues 80–104; and motif 2—PROSITE pattern PS01023—amino acid residues 173–185) are colored in red. In the amino acid sequence, putative transmembrane domains are named I to XII. Weak predicted transmembrane domains (in zebrafish PepT1a, transmembrane domains VIII and X) are colored in gray

Zebrafish *pept1a* (*slc15a1a*) cDNA was 2478 bp long, with a coding sequence (CDS) of 2154 bp encoding a putative protein of 717 amino acids (Additional file 1: Figure S1). Zebrafish PepT1a (Slc15a1a) and PepT1b (Slc15a1b) amino acid sequences shared 78% similarity and 62% identity (Fig. 1). Hydropathy analysis predicted 12 potential transmembrane domains with a large extracellular loop between transmembrane domains IX and X (Fig. 1). Structural motifs such as the PTR2 family proton/oligopeptide symporter signatures could be found in zebrafish PepT1a (Slc15a1a) sequence (amino acid residues 80–104 for signature 1, PROSITE pattern: PS0102; amino acid residues 173–185 for signature 2, PROSITE pattern: PS01023) (Fig. 1). Three putative extracellular N-glycosylation sites, one intracellular consensus region containing protein kinase C motif, and three intracellular cAMP-dependent protein kinase sequences were also identified (Additional file 1: Figure S1).

### Basic function

**Fig. 2:**
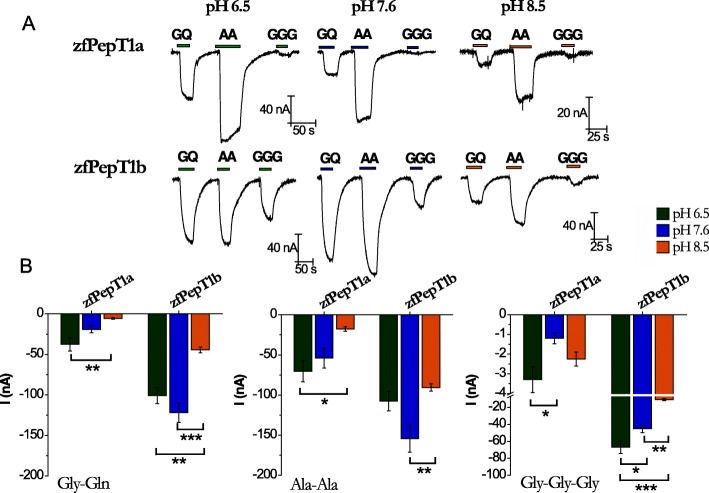
Transport activity and pH dependence of zebrafish PepT1a (Slc15a1a) and PepT1b (Slc15a1b). **a** Representative traces of transport currents in zebrafish PepT1a (zfPepT1a, top) and zebrafish PepT1b (zfPepT1b, bottom) heterologously expressed in *Xenopus laevis* oocytes. The currents in the presence of the substrates (1 mmol/L), indicated by bars, were recorded at the holding potential of − 60 mV and at pH 6.5 (left), 7.6 (middle), and pH 8.5 (right). **b** Transport-associated currents elicited by 1 mmol/L Gly-Gln (GQ) (left), Ala-Ala (AA) (middle), and Gly-Gly-Gly (GGG) (right) at − 60 mV at pH 6.5 (green), 7.6 (blue), and 8.5 (orange). Current values, shown in the histograms as the differences of the current recorded in the presence of the substrate and that in its absence, are reported as means ± SEM from 5 oocytes from 1 batch (one-way ANOVA test; *P < 0.05, **P < 0.01, and ***P < 0.001)

Figure 2 summarizes the first functional data about zebrafish PepT1a. Oocytes expressing the transporter were tested at the holding potential of − 60 mV in external control solution at pH 6.5, 7.6, or 8.5, and the substrate-induced currents (substrates: Gly-Gln, Ala-Ala, and Gly-Gly-Gly; concentration 1 mmol/L) were recorded. Representative traces for zebrafish PepT1a are in the upper part of Fig. 2a. The presence of inward currents of tens of nanoamperes amplitude clearly demonstrated that zebrafish PepT1a is electrogenic, like zebrafish PepT1b (see the lower part of Fig. 2a) and the other PepT1-type transporters so far characterized. As for the other PepT1-type transporters, the transport of zebrafish PepT1a was Na^+^-independent, regardless of the testing voltage (Additional file 1: Figure S2). In these experiments, the mean transport-associated currents of zebrafish PepT1a and PepT1b showed different profiles (Fig. 2b). In PepT1a, the amplitude of the currents (*I*) associated to the transport of the dipeptides decreased with the increase of pH from 6.5 to 8.5 (*I*_6.5_ > *I*_7.6_ > *I*_8.5_), with differences in the amplitude between the two tested pH extremes (*P* < 0.01 for Gly-Gln and *P* < 0.05 for Ala-Ala). PepT1b showed higher currents at pH 7.6 (*I*_7.6_ > *I*_6.5_ > *I*_8.5_), and the current amplitudes were different between pH 7.6 and pH 8.5 (*P* < 0.001 for Gly-Gln and *P* < 0.01 for Ala-Ala) and between pH 6.5 and pH 8.5 (*P* < 0.01 for Gly-Gln). Notably, although both transporters worked well with neutral dipeptides, PepT1a showed larger currents in the presence of Ala-Ala, while PepT1b exhibited similar current amplitudes for both substrates at the three pH tested. For the neutral tripeptide Gly-Gly-Gly, a current of ~ − 50 nA was recorded in PepT1b at pH 6.5 and pH 7.6 (with *I*_6.5_ > *I*_7.6_; *P* < 0.05), and the amplitude of current was reduced at pH 8.5 (*P* < 0.001 for pH 6.5 vs. pH 8.5, and *P* < 0.01 for pH 7.6 vs. pH 8.5). Conversely, in PepT1a, Gly-Gly-Gly elicited only very small currents regardless of the pH conditions (*P* < 0.05 for pH 6.5 vs. pH 7.6 only).

### Kinetic parameters

**Fig. 3:**
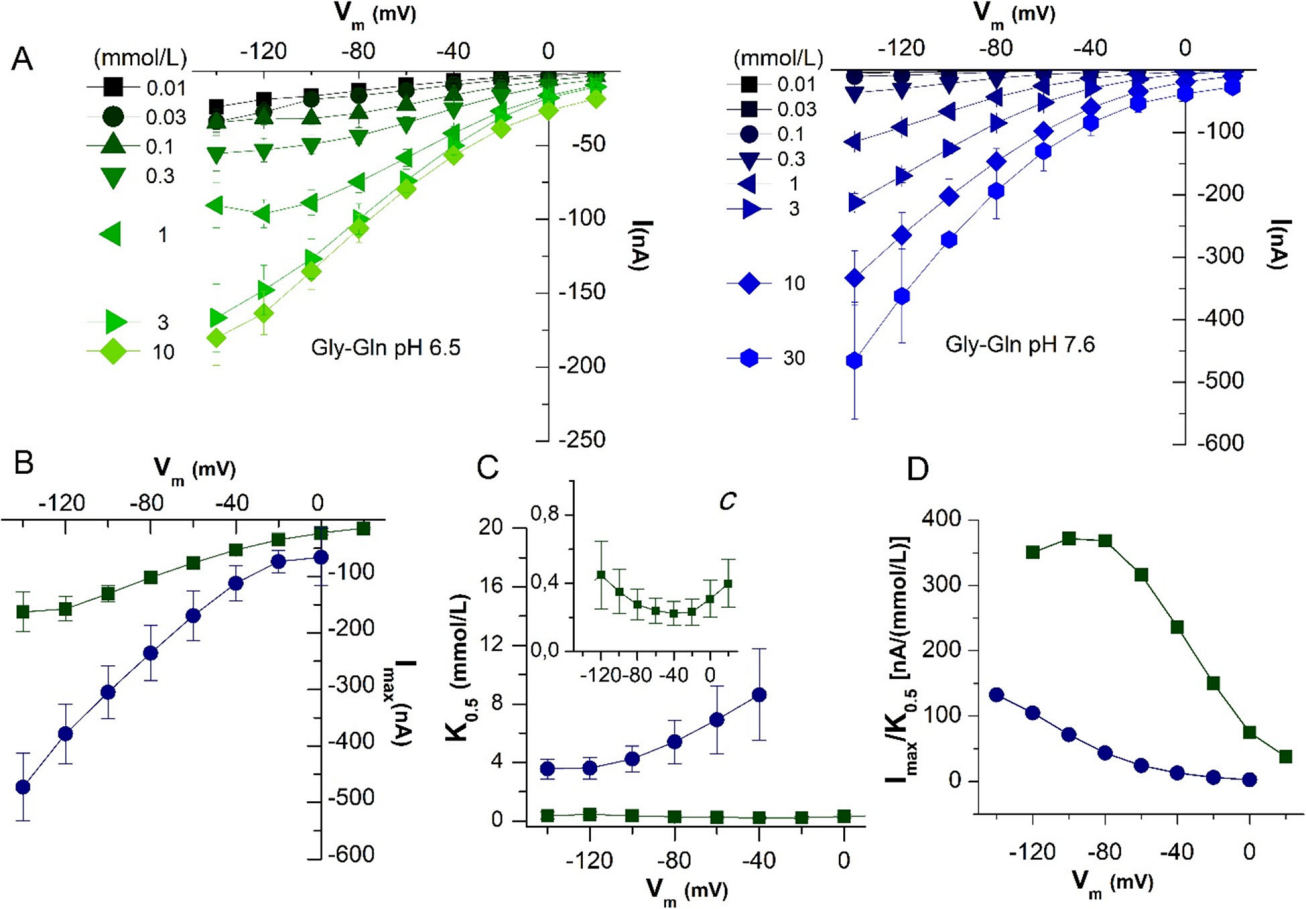
Dose-response analysis. *K*_0.5_, *I*_max_, and transport efficiency of zebrafish PepT1a (Slc15a1a) evaluated in the presence of Gly-Gln. **a** *I*/*V* relationships were obtained by subtracting the current traces in the absence to that in the presence of the indicated amounts of Gly-Gln, at pH 6.5 (green) and 7.6 (blue). The current values were fitted with the logistic equation $\left[{I}_0=\frac{-{I}_{\mathrm{max}}}{1+\left(\left[S\right]/{K}_{0.5}\ \right)}+{I}_{\mathrm{max}}\right]$ to obtain *K*_0.5_, i.e., the substrate concentration that yields one-half of the maximal current (*I*_max_), at each indicated voltage and at pH 6.5 (green square) and 7.6 (blue circle). **b** *I*_max_ at each voltage and pH. **c** *K*_0.5_ at each voltage and pH; the insert (***c***) is an enlargement of *K*_0.5_ at pH 6.5. **d** Transport efficiency, evaluated as the ratio of *I*_max_/*K*_0.5_, and plotted vs. membrane potential for the two pH conditions. *I*_max_, relative maximal current; *K*_0.5_, apparent substrate affinity; *I*_max_/*K*_0.5_, transport efficiency

To characterize zebrafish PepT1a, the currents were recorded in the presence of increasing concentrations of Gly-Gln (from 0.01 to 10 mmol/L for pH 6.5, and to 30 mmol/L for pH 7.6) and in a range of voltage from − 140 to + 20 mV (Fig. 3a). The data of transport-associated current at pH 6.5 showed that Gly-Gln 3 mmol/L is the saturating value regardless of the voltage tested. Conversely, at pH 7.6, the transport-associated current constantly increased with the substrate concentration and it did not reach the maximal value even in the presence of Gly-Gln 30 mmol/L (Fig. 3a). The *I*/*V* relationships of PepT1a at the two pH values were used to calculate the kinetic parameters, i.e., the maximal transport current (*I*_max_) and the apparent substrate affinity (i.e., the apparent concentration of peptide that yields one-half of *I*_max_; *K*_0.5_). These (Fig. 3b, c) clearly showed the changes induced in substrate interaction by increasing the pH. For example, at pH 6.5, the *I*_max_ at − 140 mV was ~ − 160 nA (*I*_max_ = − 162.79 ± 35.35 nA), but it increased close to − 500 nA (*I*_max_ = − 473.10 ± 59.89 nA) at pH 7.6. As expected, the *K*_0.5_ values differed at the two pH tested, i.e., while at pH 6.5 PepT1a showed a high affinity that did not change with the voltage (*K*_0.5_ = 0.36 ± 0.24 mmol/L at − 140 mV and *K*_0.5_ = 0.22 ± 0.07 at − 40 mV), at pH 7.6 PepT1a affinity decreased and the *K*_0.5_ values became voltage-dependent passing from ~ 3.5 mmol/L (*K*_0.5_ = 3.55 ± 0.67 mmol/L) at − 140 mV to ~ 8.5 mmol/L (*K*_0.5_ = 8.63 ± 3.12 mmol/L) at − 40 mV. Accordingly, the transport efficiency, evaluated as *I*_max_/*K*_0.5_ ratio, decreased with the increase of the pH. Also, if reported as a function of the voltage, *I*_max_/*K*_0.5_ ratios showed a completely different pattern at the two pH values (Fig. 3d), i.e., a rather complete bell-shape at pH 6.5 with a maximum at ~ − 90 mV, and a left-shift of the curve at pH 7.6 suggesting that the maximal efficiency might be reached at potentials more negative than − 140 mV. Data about *I*_max_, *K*_0.5_, and their ratio in the presence of Gly-Gln at pH 6.5 and 7.6 at the two reference membrane potentials of − 60 and − 120 mV for both PepT1a and PepT1b are summarized in Table 2.

**Table 2:** Kinetic parameters of the inwardly directed transport of Gly-Gln via the zebrafish PepT1a (Slc15a1a) and zebrafish PepT1b (Slc15a1b) measured in two-electrode voltage clamp experiments

		− 60 mV			− 120 mV			
pH	Neutral form (%)	*K*_0.5_ (mmol/L)	*I*_max_ (nA)	*I*_max_/*K*_0.5_ (nA/mmol/L)	*K*_0.5_ (mmol/L)	*I*_max_ (nA)	*I*_max_/*K*_0.5_ (nA/mmol/L)	Oocytes/batches (*n*/*N*)
PepT1a								
6.5	98.4	0.24 ± 0.07	− 75.76 ± 6.04	316.37	0.45 ± 0.19	− 157.39 ± 21.48	350.81	9/3
7.6	83.0	6.92 ± 2.34	− 169.57 ± 43.75	24.51	3.61 ± 0.73	− 378.82 ± 53.08	105.02	14/3
PepT1b								
6.5	98.4	0.13 ± 0.02	− 195.73 ± 8.89	1535.32	0.13 ± 0.02	− 396.24 ± 22.21	3032.16	7/1
7.6	83.0	2.22 ± 1.04	566.16 ± 212.44	254.54	1.01 ± 0.35	1142.30 ± 285.34	1129.67	7/1

Kinetic parameters (*K*_0.5_, *I*_max_, and *I*_max_/*K*_0.5_) were calculated on *Xenopus laevis* oocytes voltage clamped at − 60 mV and at − 120 mV and perfused with Gly-Gln in sodium chloride buffer solutions at pH 6.5 and 7.6. Values are expressed as means ± SEM of *n* oocytes (each oocyte represents an independent observation). Kinetic parameters were calculated by least-square fit to the logistic equation (Fig. 2). *I*_max_/*K*_0.5_, transport efficiency

**Fig. 4:**
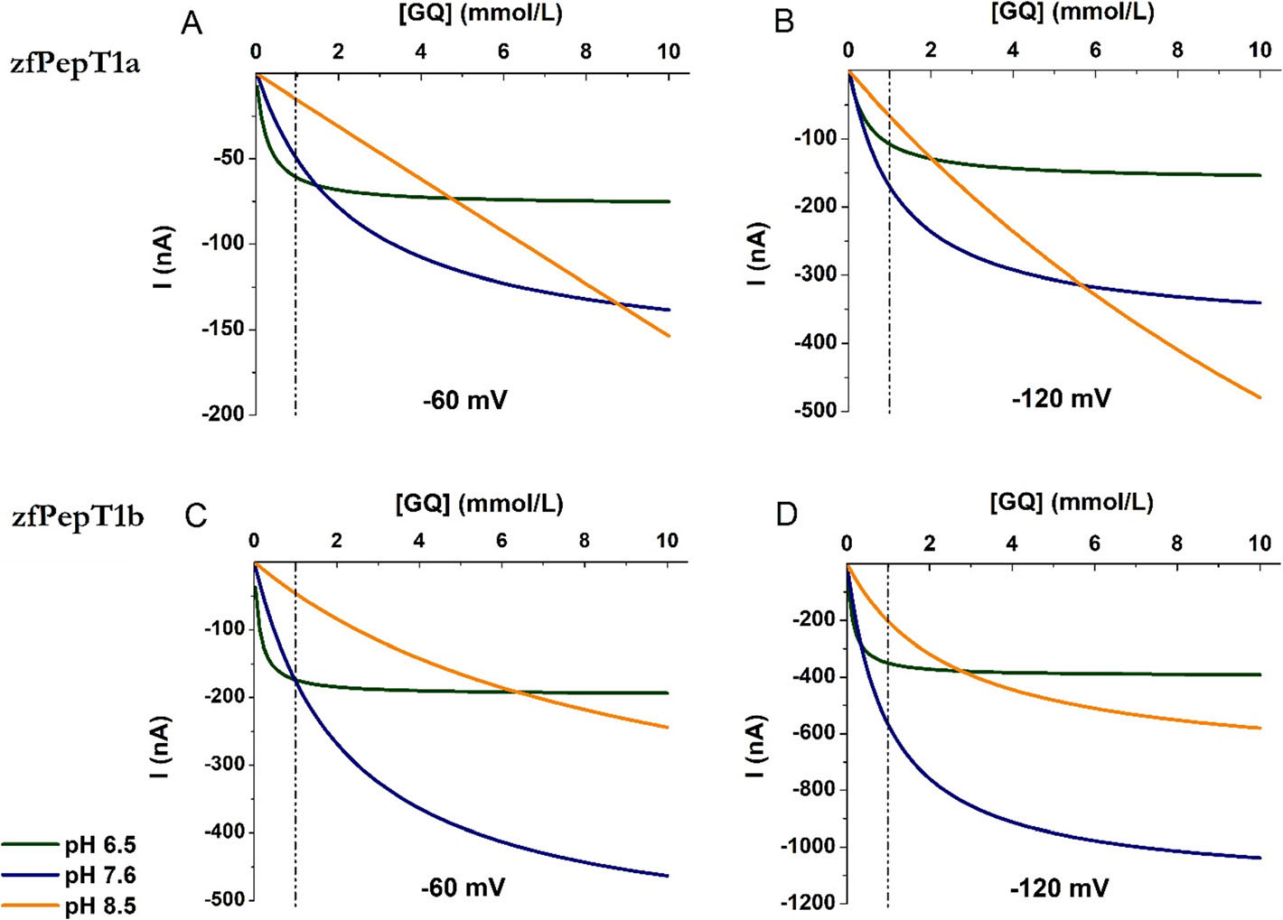
Fitting of the Gly-Gln (GQ) transport-associated currents as a function of substrate concentration (from 0.01 to 10 mmol/L) at different pH (pH 6.5 in green, pH 7.6 in blue, and pH 8.5 in orange) for two different membrane potentials: − 60 mV (left) and − 120 mV (right). **a**, **b** Zebrafish PepT1a (zfPepT1a). **c**, **d** Zebrafish PepT1b (zfPepT1b). The dashed line indicates 1 mmol/L Gly-Gln concentration

Considering the effect of the pH on the kinetic parameters and the results on the transport currents summarized in Fig. 2, the dose-response curves of Gly-Gln generated by oocytes expressing zebrafish PepT1a or PepT1b at pH 6.5, 7.6, and 8.5 were compared, and the transport-associated currents elicited by increasing concentrations of substrate from 0.01 to 10 mmol/L were plotted as current/concentration relationships and fitted with a logistic equation at − 60 and − 120 mV (see Fig. 4a, b for PepT1a and Fig. 4c, d for PepT1b). The data show that the acidic pH similarly affected the function of PepT1a and PepT1b. Due to the high affinity for Gly-Gln of both transporters at pH 6.5, the transport-associated currents reached the *I*_max_ value (see Fig. 3c for PepT1a and [14] for PepT1b) in the presence of Gly-Gln concentration lower than 10 mmol/L at the two potentials tested. At pH 7.6, the current generated by the concentration of 1 mmol/L (see Fig. 4, dashed line) is far from the maximal current in both transporters, regardless of the voltage considered. At this pH, the proteins bind the substrate with lower affinity and the current increases with increasing the concentration without showing saturation in the range of concentrations tested. At pH 8.5, the fitted curves suggest a further increase of *K*_0.5_ values, particularly for PepT1a. Notably, the transport currents elicited by concentrations of Gly-Gln up to 1 mmol/L differ of few nanoamperes only between pH 6.5 and 7.6 for both transporters at the membrane potential of − 60 mV. When the voltage is set at − 120 mV, the currents are similar at the two pH with substrate concentration up to 0.3 mmol/L, and when the substrate is 1 mmol/L at pH 7.6, the currents are larger than those at pH 6.5 for both transporters. As already reported for PepT1b [14], the curves at pH 8.5 for PepT1a suggest that the proton decrease in the external solution markedly affects the kinetic parameters, emphasizing the effects already seen at pH 7.6.

### Pre-steady-state currents

**Fig. 5:**
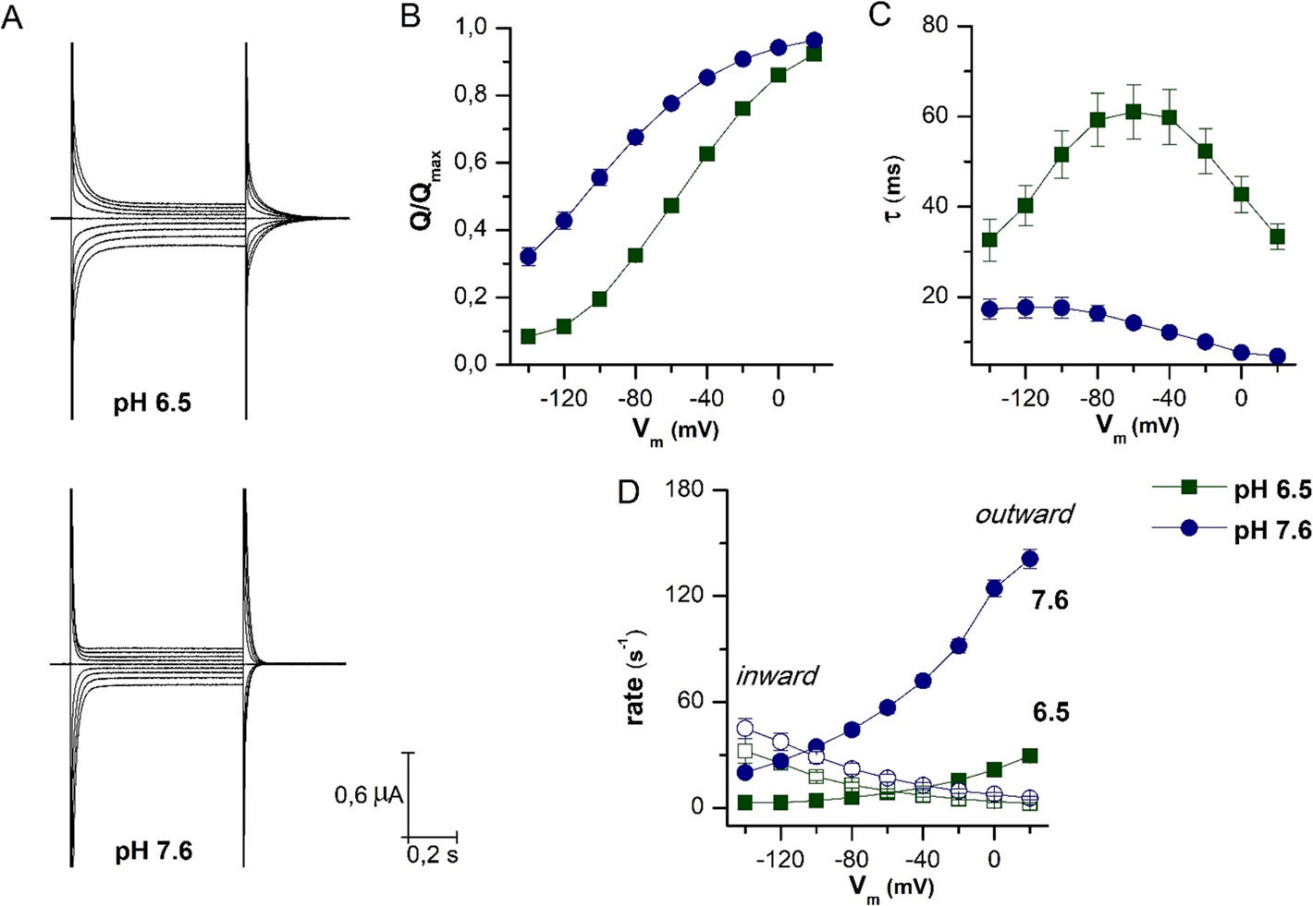
Biophysical parameters of PepT1a. **a** Representative trace of current elicited by voltage pulses in the range − 140 to + 20 mV (20 mV steps from *V*_h_ = − 60 mV) in the absence of substrate at pH 6.5 and pH 7.6 as indicated. **b**–**d** Analysis of pre-steady-state currents at pH 6.5 (green square) and 7.6 (blue circle) obtained from the slow component of a double exponential fitting of the corresponding traces in the absence of the substrate. **b** Charge/voltage (*Q*/*V*) curves obtained by integration of the pre-steady-state isolated at the two pH values. **c** Time constant/voltage (*τ*/*V*) relation; the values were estimated from the on transients, except at − 60 mV (*V*_h_), which was estimated from the off transients. **d** Unidirectional rate constants, inward (open symbols) and outward (solid symbols), of the intramembrane charge movement in function of different tested voltage conditions, derived from the *τ*/*V* relationship and the *Q*/*V* relationship at two pH conditions. Data are mean ± SEM from 10 oocytes of 3 different batches. *V*_h_, holding potential

In Fig. 5, the characteristics of the transport cycle were analyzed by investigating the behavior of the transient currents at pH 6.5 and 7.6. Pre-steady-state currents were isolated as reported in [25, 32]. From these, it is possible to calculate the total amount of charge moved in the membrane electric field, the time of relaxation decay, and the rate of outward and inward constants [28]. The representative recordings reported in Fig. 5a clearly underline the accelerating effect of reducing the number of extracellular protons. PepT1a shows the complete sigmoidal curve for the normalized *Q/V* relationship at pH 6.5 (Fig. 5b), a consistent reduction of the decay time constant (*τ*), and a complete bell-shaped curve for *τ/V* relationship (Fig. 5c). These curves are left-shifted by increasing the pH at 7.6. Consequently, the *τ* maximal value and the *V*_0.5_ move to more negative voltage. These data are very similar to those recorded in rabbit PepT1, and different to those of the zebrafish PepT1b in which *τ/V* and *Q/V* curves are left-shifted even at pH 6.5 and when the pH increases only a slight shift toward negative potentials is predicted by fitting with the Boltzmann equation. The parameters for zebrafish PepT1a, zebrafish PepT1b, and rabbit PepT1 are summarized in Table 3.

**Table 3:** Boltzmann equation parameters of zebrafish PepT1a (Slc15a1a), compared to zebrafish PepT1b (Slc15a1b) and rabbit PepT1 (Slc15a1)

	Zebrafish PepT1a		Zebrafish PepT1b		Rabbit PepT1	
pH	6.5	7.6	6.5	7.5	6.5	7.5
*Q*_max_ (nC)	41 ± 0.7	53 ± 0.6	11 ± 0.3	9.9 ± 1.1	33.2 ± 1.9	31.5 ± 1.2
*V*_0.5_ (mV)	− 57.6 ± 0.6	− 110 ± 0.7	− 108 ± 1.6	− 119 ± 7.2	− 41.4 ± 2.5	− 100 ± 2.3
*σ* (mV)	33.6 ± 0.8	39.3 ± 0.4	31.1 ± 0.9	33.5 ± 3.3	42.9 ± 3.1	39.5 ± 1.7

Boltzmann equation parameters were calculated at two pH conditions: 6.5 and 7.6 for zebrafish PepT1a (data from Fig. 5) and 6.5 and 7.5 for zebrafish PepT1b and rabbit PepT1 (data from [28]). *Q*_max_, the maximal moveable charge; *V*_0.5_, the voltage at which half of the charge is moved; *σ*, slope factor of sigmoidal curve

The *Q/V* curve represents the steady-state distribution of the transporter proteins between two conformations with the charge (intrinsic or extrinsic) in two opposite locations of the membrane electrical field. The reaction $$ {Q}_{\mathrm{in}}{\displaystyle \begin{array}{c}\overset{\mathrm{out}\mathrm{rate}}{\to}\\ {}\underset{\mathrm{in}\mathrm{rate}}{\leftarrow}\end{array}}{Q}_{\mathrm{out}} $$ describes the movement of the charge between the two positions. The outward and inward rates are the unidirectional rate constants, and *Q*_out_ and *Q*_in_ are the amount of charge respectively at the outer and inner position of the membrane electric field. Determining these rates according to [28] allows to better appreciate the effect of the pH (Fig. 5d). These data clearly show that alkalization accelerates the outward rate constant, i.e., when the pH increases to pH 7.6, the transporter completes the cycle faster, reducing the time needed for substrate translocation [33, 34].

Another piece of information coming from pre-steady-state currents records is the amount of maximal charge moved in the membrane electric field consequent to voltage steps (*Q*_max_). Zebrafish PepT1a, in the range of potentials tested, has the highest values among the tested transporters and, differently from the other PepT1 proteins, its parameters are affected by external pH (Table 3). Moreover, the slope of the *Q/V* curves (*σ*) suggests that charge movement in zebrafish PepT1a might occur over a smaller fraction of the electrical membrane field than in zebrafish PepT1b. For both transporters, when the external pH is set at 7.6, the fraction of electrical membrane field is reduced, and this effect is more evident in PepT1a.

### Tissue distribution of zebrafish pept1a (slc15a1a) in adult fish

**Fig. 6:**
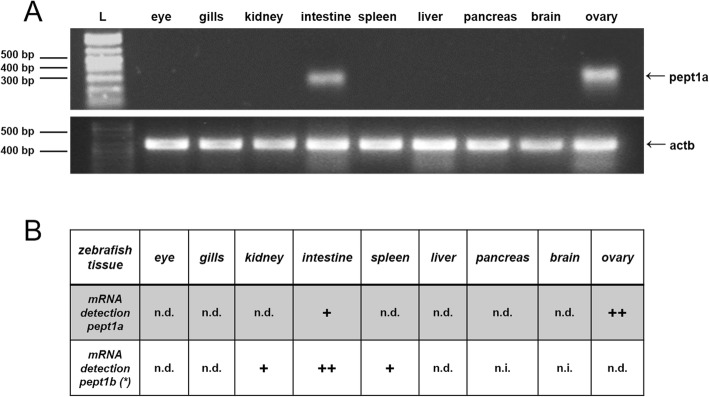
Expression analysis by RT-PCR on *pept1a* (*slc15a1a*) mRNA in adult zebrafish tissues. **a** RT-PCR assay on cDNA templates from total RNA extracted from various tissues; a PCR product of ~ 350 bp related to *pept1a* (*slc15a1a*) mRNA is present in samples from the intestine and ovary, while it is absent in the eye, gills, kidney, spleen, liver, pancreas, and brain; using the same cDNA templates, a PCR product of ~ 440 bp related to the *actb* mRNA is present in all tissue samples; L: 1 Kb Plus DNA ladder (Thermo Fisher Scientific). **b** Comparative table of *pept1a* (*slc15a1a*) vs. *pept1b* (*slc15a1b*) mRNA presence in the different zebrafish tissues analyzed. *pept1b* (*slc15a1b*) tissue expression data are from [14]. +, positive detection; n.d., not detected; n.i., not investigated

Using zebrafish *pept1a*-specific primers, a 350-bp RT-PCR product was amplified from total RNA isolated from the intestine of adult zebrafish, as well as from the ovary, while no signal was detected in the eye, gills, kidney, spleen, pancreas, and brain (Fig. 6a). As internal control to assess RNA quality, detection of β-actin (*actb*) mRNA was performed using zebrafish *actb*-specific primers, showing comparable 442-bp amplification products for all tested tissues (Fig. 6a). When investigated in the separated intestinal (rostral) bulb, mid and posterior intestine, *pept1a* (*slc15a1a*) mRNA amplification was obtained in each of the three consecutive tracts (Additional file 1: Figure S3). Notably, *pept1a* (*slc15a1a*) and *pept1b* (*slc15a1b*) were found to share the same “intestinal” localization (Fig. 6b).

### Expression of zebrafish pept1a (slc15a1a) during larval development

**Fig. 7:**
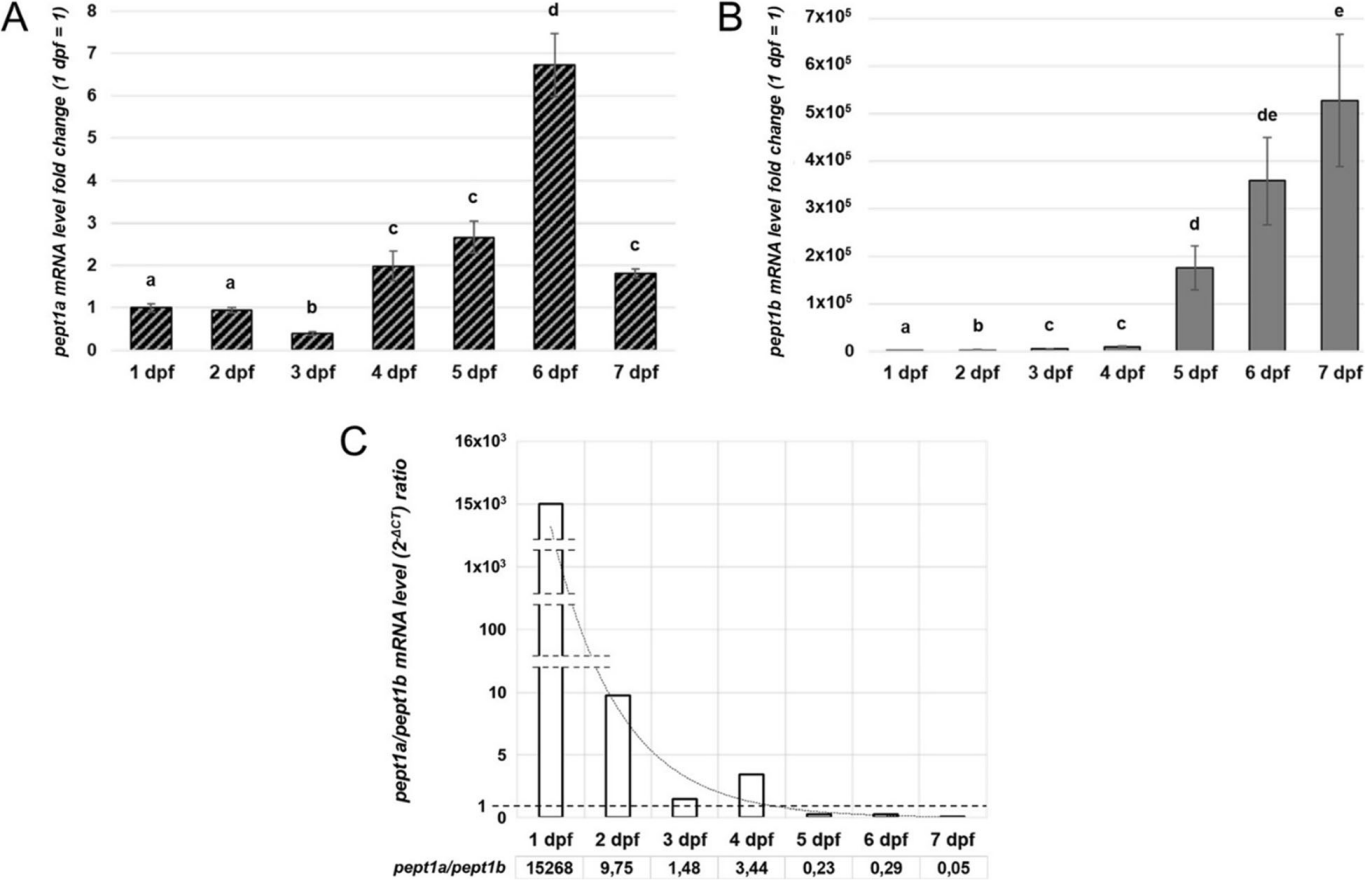
Quantitative expression analysis of zebrafish *pept1a* (*slc15a1a*) and *pept1b* (*slc15a1b*) mRNAs during early development. **a** mRNA expression analysis by qPCR in zebrafish embryos/larvae from 1 to 7 days post-fertilization (dpf). The levels of *pept1a* (*slc15a1a*) mRNA were calculated as 2^-ΔCT^ mean values obtained from two rounds of qPCR assays for each of three independent biological replicates (pools of 10–15 embryos/larvae), and then they were expressed as fold-change (*y*-axis) with respect to the 1 dpf stage taken as control value (1 dpf = 1). **b** mRNA expression analysis by qPCR of the *pept1b* (*slc15a1b*) gene in zebrafish embryos and larvae from 1 to 7 dpf. Statistical analysis of variance of the means was assessed by one-way ANOVA and Tukey's post hoc test. In histograms, different letters indicate statistically different values (*n* = 3 independent biological replicates; *P* < 0.05). **c** Representation of the trend of the *pept1a*/*pept1b* mRNA level ratio at 1 to 7 dpf, based on the 2^-ΔCT^ mean values obtained from the output data deriving from qPCR assays performed, with the same primer efficiency values, for both the *pept1a*- and *pept1b*-specific primer pairs

Zebrafish *pept1a* (*slc15a1a*) mRNA expression profile was quantitatively evaluated by qPCR during embryonic/early larval developmental stages. A polyphasic trend of mRNA expression was detected over time, passing from 1 to 7 days post-fertilization (dpf) (Fig. 7a). In particular, a negative fold-change (− 0.61) of *pept1a* (*slc15a1a*) mRNA levels was registered at 3 dpf with respect to 1 dpf (fold-change = 1), while a time-dependent increase of the signal was observed at the next stages, i.e., 4, 5, and 6 dpf (+ 2.00, + 2.66, and + 6.73 fold-change, respectively). At 7 dpf, *pept1a* (*slc15a1a*) mRNA levels were significantly reduced with respect to 6 dpf, but still slightly higher than at 1 dpf (+ 1.81). The analysis of *pept1b* (*slc15a1b*) mRNA levels in the same developmental stages revealed a time-dependent trend with a strong increase from 1 up to 7 dpf (Fig. 7b). In detail, a very faint signal for zebrafish *pept1b*-specific mRNA was detected at 1 dpf (fold-change = 1), which increased ~ + 1.5 × 10^3^-fold already at 2 dpf, reaching ~ + 8.8 × 10^3^- at 4 dpf and + 5.3 × 10^5^-fold increase at 7 dpf. A relative comparison of the expression level data (2^-ΔCT^ values) was inferred by calculating the *pept1a*-to-*pept1b* ratio at each developmental stage. The ratio between the expression levels was > 1 from 1 to 4 dpf and < 1 from 5 to 7 dpf (Fig. 7c).

## Discussion

Like many other animal models, zebrafish is a highly tractable organism and despite being a vertebrate it offers an extremely high potential for genetic analysis and cellular observation. Also, it is readily available and easy to breed, and its transparent embryo develops quickly. The zebrafish “model system” now comprises a sequenced genome, thousands of mutants, transgenic tools, staging series, know-how for imaging, embryological manipulation, drug discovery, and a lot more (among many other papers see, e.g., [24, 35]; among many other excellent reviews see, e.g., [36–38]). However, to join the rank of top model organism for biomedical research, the suite of tools and resources already available needs to be implemented with robust phenotyping and functional analyses at the same performance levels of investigation and with the same advanced experimental approaches and methods as those considered standard for mammalian models (e.g., rodents) and humans. Searching in detail for (a) species-specific molecular phenotype(s) offers the possibility to define how many orthologous/paralogous proteins from same/different species operate similarly or differently, from one another, thus opening to the comprehension of the multiplicity of structural-functional solutions at the molecular level in the various animal bio-systems analyzed. This is particularly true for epithelial physiology, and in transport and transporters functional analyses, in which the complexity of the substrate specificities largely meets the complexity of the model species. Thus, in the context of this discussion, which evaluates the zebrafish as a suitable model organism in nutrigenomics and nutrition research, emphasis should be given to the concept that a very careful evaluation of the impact of a study on (a) membrane transporter(s) in a non-human/non-rodent model system is always required (see, e.g., [39]).

In this “case” study, we report the functional characterization of the zebrafish PepT1a-type transporter, which completes the functional picture of the triad of PepT-type transporters, i.e., PepT1a (Slc15a1a), PepT1b (Slc15a1b), and PepT2 (Slc15a2), expressed by the zebrafish (for details, see Table 1). This study confirms and extends results recently obtained in the Atlantic salmon [31] and leads to the general assumption that PepT1a-type transporters physiologically operate in teleost fish. In particular, zebrafish *pept1a* (*slc15a1a*), which is expressed in the intestinal tract and in the ovary of adult fish, generates a protein product, i.e., PepT1a (Slc15a1a), that is able to mediate the transport of neutral di/tripeptides, such as Gly-Gln, Ala-Ala, and Gly-Gly-Gly. However, PepT1a (Slc15a1a) differs from the already well-characterized zebrafish PepT1b (Slc15a1b) in terms of transport kinetics, substrate specificity, and transport efficiency. Notably, *pept1a* (*slc15a1a*) mRNA expression profile during the first 7 dpf also differs from the *pept1b* (*slc15a1b*) [14], highlighting a possible role of *pept1a* (*slc15a1a*) mRNA in the first 3 days of embryonic development and as a component of the maternal mRNA pool. Whether or not *pept1a* (*slc15a1a*) plays a physiological role in extra-embryonic tissue(s), such as the yolk syncytial layer, remains a relevant, yet unanswered, question.

### Function

The basic transport currents recorded (and reported in Fig. 2 and Additional file 1: Figure S2) confirm that zebrafish PepT1a (Slc15a1a), like the recently characterized Atlantic salmon PepT1a (Slc15a1a) [31], is electrogenic and capable of transporting di/tripeptides in a H^+^-dependent manner and independently of the presence of sodium ions. However, while eliciting transport currents, the 1 mmol/L substrate condition is not the most adequate to test the pH dependence of the transport because of the considerable effects of pH on substrate affinity. Nevertheless, the representative traces in Fig. 2a and the analysis of the transport-associated currents in Fig. 2b clearly indicate that the differences in amino acid sequences between the two transporters may have functional implication(s) in both pH dependence and substrate preferences. In fact, if compared to the well-characterized zebrafish PepT1b (Slc15a1b), PepT1a (Slc15a1a) (i) prefers Ala-Ala to Gly-Gln, (ii) works well at acidic pH, and (iii) gives rise to relatively smaller currents in all conditions tested. Moreover, Gly-Gly-Gly appears to be a poor substrate for this transporter. When the transport-associated currents are recorded in the presence of increasing concentrations of Gly-Gln by using a standard step protocol, PepT1a (Slc15a1a) shows some common features with PepT1b (Slc15a1b), but also its own. In particular, the pH has a large effect on the affinity of the PepT1a transporter, which is evident by observing the behavior of the *I*/*V* curves reported in Fig. 3 and comparing the *K*_0.5_ and *I*_max_ values of the PepT1a and PepT1b proteins (Table 2). At pH 6.5, both proteins work similarly, as it results when considering the data reported for − 120 and − 60 mV in Fig. 4 and Table 2, but when the pH increases to 7.6, the two proteins work differently, and PepT1a affinity for Gly-Gln is largely influenced by pH. For instance, when the external pH is set at 7.6, the amount of substrate to reach one-half of the maximal currents (*K*_0.5_) increases in PepT1a [e.g., at − 60 mV, *K*_0.5_ is ~ 6.92 mmol/L at pH 7.6 and ~ 0.24 mmol/L at pH 6.5 (ratio 28.83)] more than it occurs in PepT1b [e.g., at − 60 mV, *K*_0.5_ ~ 2.22 mmol/L at pH 7.6 and ~ 0.13 mmol/L at pH 6.5 (ratio 17.07)]. This suggests that PepT1a has very strong pH dependence, which is confirmed by data reported in Fig. 4 where the fitting dose-response of the two transporters is compared.

The pre-steady-state currents are elicited by voltage steps and are due to the charges moved inside the membrane electric field. In many solute carriers, they are recordable and related to the first steps of the transport cycle. These currents are due to one or more intrinsic (internal, that is one or more protein residues, i.e., charged amino acids) or extrinsic (external, that is ion or proton) charges, present or entered in the membrane electric field that are moved by voltage changes. As suggested by the analysis of the pre-steady-state currents, the main effects of protons are on the turnover rate of the transporter (1/*τ*), i.e., the two proteins cycle differently and are differently affected by the pH. In response to voltage steps, the amount of charges moved by PepT1a is higher, while the time decay of pre-steady-state currents and the unidirectional rate constants are lower (slower) if compared to PepT1b [28]. At pH 6.5, the *τ*/*V* relationship for PepT1a shows a complete bell-shape with the slower value at − 60 mV, like the rabbit PepT1 reported in [28]. This curve is reduced and left-shifted at pH 7.6. In PepT1b, the curves are left-shifted and faster even at pH 6.5 and changing the pH decreases *τ* values and only slightly moves the curve to a more negative potential. In this transporter, the slower value for *τ* is recorded at pH 6.5 at − 140 mV. For PepT1a, the *Q*/*V* relationship is a complete sigmoidal curve in the range of voltage tested, confirming the symmetrical behavior of the transient current at this pH for PepT1a. As for other PepT1 transporters, also for zebrafish PepT1a with the increase of the pH both *Q*/*V* and *τ*/*V* curves shift to more negative voltage, increasing the transport rate (1/*τ*). The effect of protons is evident on the unidirectional rate constants, with a main effect on the outward rate, which greatly increases its value by changing the pH (from ~ 30 s^−1^ at pH 6.5 to ~ 140 s^−1^ at pH 7.6, at + 20 mV).

Summarizing the data from the kinetic parameters and the pre-steady-state currents, we suggest that zebrafish PepT1a and PepT1b residues involved in protons and substrates binding/interaction are differently located in the membrane electric field and that the two transport proteins are differently affected by changes in external proton concentration. Consequently, the pH might alter in a different way the steps of transport cycle differently influencing the transport rate of the two proteins. Interestingly, PepT1a shows a “mammalian” behavior [28, 34]. In addition, the data collected on the biophysical parameters make it more similar to rabbit PepT1 than to the other fish transporters. To date, we have not been able to identify any obvious amino acid residues along the primary sequences and/or in the three-dimensional structures deposited in databanks that can possibly be associated to protons and/or substrates binding/interaction. However, a systematic analysis, mainly based on site-directed mutagenesis of amino acid residues selected by means of sequence and structure coevolution computational criteria (see, e.g., [40]) and subsequent electrophysiological analysis in oocytes, will answer this question.

### Expression

Similar to recent findings in Atlantic salmon [31], the analysis of the expression profile clearly shows that zebrafish *pept1a* (*slc15a1a*) mRNA is present in the intestine of the adult fish. In particular, zebrafish *pept1a* (*slc15a1a*) mRNAs are detected in the intestinal bulb, mid and posterior intestinal segments (see Additional file 1: Figure S3). In this respect, zebrafish *pept1a* (*slc15a1a*) partially overlaps zebrafish *pept1b* (*slc15a1b*) since the latter is very strongly expressed in the proximal intestine of this teleost fish (see, e.g., [12–14]). However, unlike the Atlantic salmon [26, 31], further and ad hoc studies are still needed to precisely define the relative mRNA amounts of *pept1a* (*slc15a1a*) alone and vs. *pept1b* (*slc15a1b*) in the various segments of the alimentary canal of the adult zebrafish.

During early development (1–7 dpf), *pept1a* (*slc15a1a*) mRNA expression seems to undergo a multi-phasic trend over time. The mRNA levels already present (in the ovary of the adult fish and thus in the unfertilized eggs and) at 1 and 2 dpf, possibly due to the occurrence of *pept1a* (*slc15a1a*) mRNA in the maternal RNA pool, significantly decrease at 3 dpf. Interestingly, recovery and significant increase of mRNA levels are measured from 4 to 6 dpf, which is in line with *pept1a* (*slc15a1a*) baseline expression as extrapolated from a recent transcriptional profiling (high-resolution mRNA expression time course) of zebrafish embryonic developmental stages [35]. It is worth noting that this phenomenon parallels maturation of the gut and achievement of the full digestive/absorptive function (see, e.g., [41, 42]), during which *pept1a* (*slc15a1a*) mRNA new-synthesis seems to occur. Remarkably, a third phase of expression is hinted by the reduced levels of *pept1a* (*slc15a1a*) at 7 dpf with respect to 6 dpf, which suggests a time frame-specific functional expression of *pept1a* (*slc15a1a*) that needs to be further addressed. This *pept1a* (*slc15a1a*) expression trend during development appears even more interesting when compared to the qPCR expression data for the *pept1b* (*slc15a1b*) mRNA levels which, as expected (see, e.g., [12–14]), are found to increase day by day strongly and progressively, starting from the very faint signal at 1 dpf and then increasing to more than 5 × 10^5^-fold at 7 dpf. Assuming that the “quantitative” comparative evaluation of the *pept1a* (*slc15a1a*) vs. *pept1b* (*slc15a1b*) expression levels goes beyond the aim of this paper and the analyses in question, we cannot but noticing that the “raw” calculation (based on 2^-ΔCT^ values) of the *pept1a* (*slc15a1a*)-to-*pept1b* (*slc15a1b*) expression ratio seems to indicate that *pept1a* (*slc15a1a*) expression prevails on *pept1b* (*slc15a1b*) during the 1-to-4 dpf period, while the expression level ratio becomes lower than 1 from 5 dpf on, hinting that *pept1a* (*slc15a1a*) may be the predominant *pept1*-type mRNA species at the immediacy over time. Whether or not this expression trend is general or zebrafish-specific remains an open question. In fact, at least to our knowledge, the only study available in the literature that specifically compares *pept1a* (*scl15a1a*) and *pept1b* (*slc15a1b*) in pre-feeding stages larvae refers to the Mozambique tilapia (*Oreochromis mossambicus*) and is limited to the intestinal organ only. In this case, *pept1a* (*slc15a1a*) and *pept1b* (*slc15a1b*) temporal trend of expression in the intestine goes parallel from 3 to 14 dpf (for details, see Table 4, and literature therein [49]).

**Table 4:** Organ/tissue distribution of *pept1a* (*slc15a1a*) and *pept1b* (*slc15a1b*) mRNA in teleost fish species for which the expression of the two genes has contemporarily been studied. Whenever co-analyzed *pept2* (*slc15a2*) mRNA expression has also been considered

Species [order]	Developmental stage	Description	GenBank Acc. No.	Organ/tissue distribution (observed in the study)	Distribution along the post-gastric alimentary canal (observed in the study)	Notes	References
Mummichog (*Fundulus heteroclitus*) [Cyprinodontiformes]	Adults (~ 9 g)	*pept1a* (*slc15a1a*)	JN615008.1	Intestine	Anterior intestine ≈ posterior intestine	Environmental (freshwater acclimation vs. seawater acclimation) and nutritional (fasting vs. re-feeding) regulation of *pept1a* (*slc15a1a*) and *pept1b* (*slc15a1b*)	[43]
		*pept1b* (*slc15a1b*)	JN615007.1		Anterior intestine ≈ posterior intestine		
Nile Tilapia (*Oreochromis niloticus*) [Cichliformes]	Juveniles (~ 12 g)	*pept1a* (*slc15a1a*)	XM_005452882	Intestine >>> stomach > brain > gill > liver	Proximal intestine >> mid intestine >>> distal intestine	Environmental (waterborne copper exposure) and/or nutritional (fasting vs. re-feeding) regulation of *pept1a* (*slc15a1a*), *pept1b* (*slc15a1b*) and *pept2* (*slc15a2*)	[44]
		*pept1b* (*slc15a1b*)	XM_005465251	Intestine >>> brain ≈ stomach	Mid intestine > proximal intestine >>> distal intestine		
		*pept2* (*slc15a2*)	XM_005475385	Intestine >> stomach > kidney > liver ≥ gill ≈ brain > spleen > muscle	Mid intestine >>> distal intestine > proximal intestine		
	Adults (~ 62 g)	*pept1a* (*slc15a1a*)	XM_013267250.1	Intestine	Anterior intestine > middle intestine >>>> posterior intestine	Environmental (high-salinity acclimation) regulation of *pept1b* (*slc15a1b*)	[45]
		*pept1b* (*slc15a1b*)	XM_005452882.2		Anterior intestine ≈ middle intestine >>>> posterior intestine		
		*pept2* (*slc15a2*)	XM_005475385		Posterior intestine > middle intestine >> anterior intestine		
	Adults (~ 125 g)	*pept1a* (*slc15a1a*)	XM_003459630	Intestine	Anterior intestine > middle intestine >>> posterior intestine	Nutritional (dietary salt supplementation) regulation of *pept1a* (*slc15a1a*), *pept1b* (*slc15a1b*) and *pept2* (*slc15a2*)	[46]
		*pept1b* (*slc15a1b*)	XM_003447363		Middle intestine > anterior intestine >>> posterior intestine		
		*pept2* (*slc15a2*)	XM_003454878		Posterior intestine ≥ middle intestine >> anterior intestine		
Mozambique tilapia (*Oreochromis mossambicus*) [Cichliformes]	Adults (~ 97 g)	*pept1a* (*slc15a1a*)	XM_003459630	Intestine	Anterior and middle intestine	Environmental (salinity-dependent) nutritional regulation of *pept1a* (*slc15a1a*), *pept1b* (*slc15a1b*) and *pept2* (*slc15a2*)	[47]
		*pept1b* (*slc15a1b*)	XM_003447363		Anterior and middle intestine		
		*pept2* (*slc15a2*)	XM_003454878		Middle and posterior intestine		
	Adults (100–250 g)	*pept1a* (*slc15a1a*)	LC197343	Intestine	Hepatic loop > proximal major coil >>> gastric loop ≈ distal major coil ≈ terminal segment	Nutritional (fasting vs. re-feeding) regulation of *pept1a* (*slc15a1a*)	[48]
	Adults (~ 24 g)	*pept1a* (*slc15a1a*)	XM_013267250.1	Intestine	Anterior intestine > middle intestine >>>> posterior intestine	Environmental (high-salinity acclimation) regulation of *pept1a* (*slc15a1a*) and *pept2* (*slc15a2*)	[45]
		*pept1b* (*slc15a1b*)	XM_005452882.2		Anterior intestine ≈ middle intestine >>>> posterior intestine		
		*pept2* (*slc15a2*)	XM_005475385		Posterior intestine > middle intestine >> anterior intestine		
	Adults (~ 54 g)	*pept1a* (*slc15a1a*)	KX034112.1	Intestine >>>> pituitary ≈ skin ≈ muscle ≈ kidney ≥ heart ≈ brain ≈ gills ≥ liver ≈ fat ≥ stomach ≈ esophagus ≈ spleen	Anterior intestine >>> middle intestine >> posterior intestine	–	[49]
		*pept1b* (*slc15a1b*)	KX034110.1	Intestine >>>> brain > pituitary > muscle > skin ≈ gills ≈ heart ≈ liver ≈ fat ≈ spleen ≈ kidney ≈ esophagus ≈ stomach	Anterior intestine >>>> middle intestine > posterior intestine		
		*pept2* (*slc15a2*)	KX034111.1	Intestine ≥ kidney >> muscle ≥ liver > brain ≈ pituitary ≈ skin ≈ stomach > heart ≈ spleen ≈ heart > gills	Middle intestine >>>> posterior intestine >> anterior intestine		
	Pre-feeding larvae (3–14 dpf)	*pept1a* (*slc15a1a*)	KX034112.1	Intestine	Whole intestine	Temporal (time course from the pre-hatching to completion of yolk sac resorption stage) regulation of *pept1a* (*slc15a1a*), *pept1b* (*slc15a1b*) and *pept2* (*slc15a2*)	
		*pept1b* (*slc15a1b*)	KX034110.1				
		*pept2* (*slc15a2*)	KX034111.1				
European seabass (*Dicentrarchus labrax*) [Perciformes]	Juveniles (~ 1.2 g)	*pept1a* (*slc15a1a*)	–	Intestine	Whole intestine	Environmental (short- and long-term low-salinity acclimation) regulation of *pept1a* (*slc15a1a*), *pept1b* (*slc15a1b*) and *pept2* (*slc15a2*)	[50]
		*pept1b* (*slc15a1b*)	–				
		*pept2* (*slc15a2*)	–				

Assayed by quantitative real-time PCR, *dpf* days post-fertilization

Understanding the functional importance, and thus the physiological implications, of having two similar transporters that work in the same biological district(s) with dissimilar kinetics and possibly dissimilar expression levels is a major topic of peptide transport research in teleost fish, with promising implications in higher vertebrate and human physiology. In the case of our PepT1-type transporters, the expression data bring our attention to both the intestine and the yolk syncytial layer.

The presence of PepT1a and PepT1b in the intestine has been related to the possible variability of the natural environment where the fish live, to the nutritional input and to the peculiarities of the digestive system of the various fish species and to a variety of other challenges (reviewed in [12, 13]). In particular, in a large number of teleost fish species, zebrafish and other cyprinids included, the spatio-temporal expression of PepT1b intestinal mRNA largely varies during ontogeny, in response to nutritional states (e.g., food deprivation/re-feeding), dietary challenges, and/or environmental conditions (e.g., in freshwater/seawater adaptation), as well as under certain disease states (e.g., gut inflammation) [14–22, 26, 30, 51–63]. But, in the light of the most recent findings, the new view that PepT1a and PepT1b may both be expressed and operate in teleost fish models and similarly or differently respond to the various internal and external solicitations should always be taken into account (for details, see Table 4, and literature cited therein [43–48, 50]).

Moreover, the findings during zebrafish early development open to novel interesting scenarios in applied nutrition and nutrigenomics. In fact, in a perspective, the expression of specific genes involved in nutrients utilization, such as *pept1a* (*slc15a1a*), if located at the level of the yolk sac structures might become functional to the systematic comprehension of the uptake processes of all the nutritional resources in it contained. In particular, the presence of selected transporters that operate with their kinetic properties on selected and rather homogenously represented yolk protein degradation products (e.g., those from vitellogenin, phosvitin, and lipovitellin; see, e.g., [64, 65]) might be highly informative to fully understand the rules and the dynamics of the proteolysis process(es) as a whole. In addition, it could help to address specifically the fate of a variety of highly relevant nutritional, immunological, and/or differently bioactive peptides there generated.

## Conclusions

Molecular cloning and functional expression in a heterologous system has allowed the characterization of a second PepT1-type transporter, after PepT1b *alias* PepT1, in the zebrafish. The zebrafish represents the second PepT1a-type transporter, after the Atlantic salmon, for which thorough functional characterization by two-electrode voltage clamp (TEVC) has been achieved. Therefore, the concept that PepT1a does functionally act in teleost fish model systems can be fully asserted. In this context, re-evaluation of the di/tripeptide absorptive model along the alimentary canal of teleost fish (for review see, e.g., [12–14]) should be considered in the light of the fact that two PepT1-type transporters—and not one like in higher vertebrates such as mammals and birds—operate at the intestinal level. Whether or not PepT1a and PepT1b transporters share physiological roles, cellular localization in the intestinal epithelium, sub-cellular localization in the intestinal epithelial cells, type of regulation, etc. are questions to be addressed, but in this respect, the zebrafish model and its toolbox represent the most suitable teleost fish experimental system to answer these questions. All together, the molecular and functional data obtained for zebrafish (and Atlantic salmon) PepT1a, together with the molecular and functional data already available and extended from zebrafish (and Atlantic salmon) PepT1b, allow combinatorial analysis of kinetics properties vs. primary amino acid sequences, which might help in identifying specific amino acids along the primary sequences relevant for substrate specificity, pH dependence, transport efficiency, turnover rate, etc. Comparison studies with higher vertebrate orthologs, such as human PEPT1 (SLC15A1) and murine PepT1 (Slc15a1), might be translational to human physiology and pharmacology, and in the context of this discussion in nutrigenomics, dietetics, and nutrition research, and in developing new model(s) of substrate-transporter interaction(s), pharmacophore(s), etc. In this respect, this set of PepT1a- plus PepT1b-type transporters from teleost fish may represent an original tool to support structure-function studies at the molecular level. Moreover, there are several pieces of evidence suggesting that PepT1-type proteins operate in the membrane in oligomeric (tetrameric) state (for review, see, e.g., [13]). Were they co-expressed in the same cell type, PepT1a- and PepT1b-type transporters could form hetero-tetramers and possibly interact cooperatively for optimal di/tripeptide transport function. Another functional consideration regards the question of whether or not teleost fish PepT1-type transporters are linked to any Na^+^/H^+^ exchanger(s) at the apical membrane of the enterocyte, like it occurs in the mammalian systems where the antiporter plays a major role in building the inwardly directed H^+^-gradient that supports H^+^-dependent peptide uptake (see, e.g., [66, 67]). The comparison between agastric, such as the zebrafish, and gastric, such as the Atlantic salmon, teleost fish models might help going more systematically into the details of such a physiological question departing from the singularity of the zebrafish model (see, e.g., [12–14]). Last but not least, due to its expression during the early embryonic development, it has to be fully considered the hypothesis that zebrafish *pept1a* (*slc15a1a*) (like other solute carriers involved in sugar, lipid, amino acid, anion, and metal ion uptake [68–74]) is part of the maternal machinery that supports early developmental stages and/or it is expressed in an extra-embryonic tissue such as the yolk syncytial layer. If so, it could operate in specifically mediating the uptake of the di/tripeptides that derive from the yolk protein degradation processes, thus strategically contributing to provide the bulk of protein nitrogen for early embryo development and growth. If so, the kinetic properties of *pept1a* (*slc15a1a*) would well support yolk protein uptake in the embryonic and larval zebrafish, making *pept1a* (*slc15a1a*) a specific marker of the yolk protein degradation process.

## Methods

### Animals

Zebrafish (wild-type AB) were maintained and bred at the High Technology Centre (HIB), Department of Biological Sciences, University of Bergen, according to standard protocols as described elsewhere [75]. Zebrafish embryos were obtained from natural mating. The developing embryos/larvae were incubated at 28.5 °C until use. Developmental stages of zebrafish embryos/larvae were expressed as dpf at 28.5 °C [76].

Adult fish were anesthetized by immersion in 0.2 g/l MS-222 and then killed by decapitation prior to organ removal and dissection. Developing embryos/larvae were euthanized by anesthetic overdose before sampling into RNALater (Qiagen, Hilden, Germany).

### Molecular cloning

Zebrafish *pept1a* (*slc15a1a*) gene sequence was retrieved from the Genome Data Viewer (GDV) tool at the NIH U.S. National Library of Medicine (NCBI) from the zebrafish GRCz11 Genome Assembly (RefSeq Acc. No. GCF_000002035.6; GenBank Acc. No. GCA_000002035.4; submitter: Genome Reference Consortium; annotation release 106; release date 26 June 2017), where it is located on chromosome (Chr) 9: 1,136,369-1,163,151 (GenBank Acc. No. NC_007120.7). To amplify *pept1a* (*slc15a1a*), specific primers were designed on the genomic sequence (GenBank Acc. No. NC_007120.7), in the untranslated regions (UTR) flanking the CDS upstream exon 1 (5′ UTR) and downstream exon 24 (3′ UTR) (Additional file 1: Table S1). Total RNA was isolated from zebrafish intestine as described below. cDNA was synthesized from 5 μg of total RNA using SuperScript III First-Strand Synthesis system for RT-PCR kit (Thermo Fisher Scientific, Monza, Italy) with Oligo (dT) primers according to the manufacturer’s protocol. *pept1a* (*slc15a1a*) cDNA was amplified using specific primers and Platinum® Taq DNA Polymerase High Fidelity (Thermo Fisher Scientific) according to the manufacturer’s protocol, with a T100™ Thermal Cycler (Bio-Rad). PCR products were checked on 1% (w/v) agarose gel, purified using QIAquick Gel Extraction Kit (Qiagen), and cloned into a StrataClone blunt PCR cloning vector pSC-B (Agilent Technologies, La Jolla, CA, USA) following the manufacturer’s protocol. Sequencing was performed at the University of Insubria (Varese, Italy), and sequence identity was confirmed by tBLASTx analysis against the GenBank database.

### Sequence analysis

Pairwise alignment of zebrafish PepT1a and PepT1b protein sequences was performed using Clustal Omega (https://www.ebi.ac.uk/Tools/msa/clustalo/) [77] with default parameters (Gonnet series matrix, Gap opening penalty 10, Gap extension 0.2). Alignment was displayed in GeneDoc 2.7 software [78], and the percentage of sequence identity and similarity between the paralogue proteins calculated. The putative transmembrane domains were predicted using TMHMM v. 2.0 program as implemented in SMART (http://smart.embl-heidelberg.de/) [79, 80]. Potential N-glycosylation sites, cAMP/cGMP-dependent protein kinase phosphorylation sites, and protein kinase C phosphorylation sites were predicted using the ScanProsite tool (https://prosite.expasy.org/scanprosite/) [81].

### Expression in *Xenopus laevis* oocytes and electrophysiology

The full length of cDNA encoding zebrafish PepT1a was subcloned in pSPORT1 for *Xenopus laevis* oocyte expression. The construct was verified by sequencing.

The recombinant plasmids (pSPORT1-zfPepT1a) were linearized with NotI and purified with Wizard SV Gel and PCR clean-up system (Promega Italia, Milan, Italy), in vitro capped and transcribed using T7 RNA polymerase. The purified cRNA was quantified by NanoDrop™ 2000 Spectrophotomer (Thermo Fisher Scientific). All enzymes used were supplied by Promega Italia.

The oocytes were obtained by laparotomy from adult female *Xenopus laevis* (Envigo, San Pietro al Natisone, Italy). The frogs were anesthetized by immersion in MS222 0.10% w/v solution in tap water adjusted at final pH 7.5 with bicarbonate, and after the treatment with an antiseptic agent (povidone-iodine 10%), the frog abdomen was incised and the portions of the ovary removed. The oocytes were treated with 1 mg/mL collagenase (Sigma Collagenase from *Clostridium histolyticum*) in calcium-free ND96 (NaCl 96 mmol/L, KCl 2 mmol/L, CaCl_2_ 1.8 mmol/L, MgCl_2_ 1 mmol/L, HEPES 5 mmol/L, pH 7.6) for at least 1 h at 18 °C. The healthy and full-grown oocytes were selected and separated manually in NDE solution (ND96 plus 2.5 mmol/L pyruvate and 0.05 mg/mL gentamycin sulphate). After 24 h at 18 °C, the oocytes were injected with 25 ng (in 50 nL of water) of in vitro synthesized zebrafish PepT1a cRNA using a manual microinjection system (Drummond Scientific Company, Broomall, PA, USA). Before electrophysiological studies, the oocytes were incubated at 18 °C for 3–4 days in NDE [82].

The membrane currents under voltage clamp conditions controlled by Clampex 10.2 (Molecular Devices, Sunnyvale, CA, USA) were recorded by TEVC (Oocyte Clamp OC-725C, Warner Instruments, Hamden, CT, USA). The electrodes, with a tip resistance of 0.5–4 MΩ, were filled with 3 mol/L KCl. Bath electrodes were connected to the experimental oocyte chamber via agar bridges (3% agar in 3 mol/L KCl). The holding potential was kept at − 60 mV; the voltage pulse protocol consisted of 10 square pulses from − 140 to + 20 mV (20 mV increment) of 700 ms each. Signals were filtered at 0.1 kHz and sampled at 200 Hz or 0.5 kHz and at 1 kHz. Transport-associated currents were calculated by subtracting the traces in the absence of substrate from those in its presence. Data was analyzed using Clampfit 10.7 (Molecular Devices). Transient currents were analyzed using double exponential methods in order to separate the endogenous capacitive component of the oocytes. The equilibrium distribution of the charge moved during the pre-steady-state currents was fitted with the Boltzmann equation:

$$ Q=\frac{Q_{\mathrm{max}}}{1+\exp \left[\frac{-\left(V-{V}_{0.5}\right)}{\sigma}\right]} $$

where *Q*_max_ is the maximal moveable charge, *V*_0.5_ is the voltage at which half of the charge is moved (that is, the midpoint of the sigmoidal), and *σ* = *kT/qδ* represents a slope factor, in which *q* is the elementary electronic charge, *k* is the Boltzmann constant, *T* is the absolute temperature, and *δ* is the fraction of electrical field over which the charge movement occurs [28]. All figures were prepared with Origin 8.0 (OriginLab, Northampton, MA, USA). The external control solution had the following composition: NaCl (or TMA) 98 mmol/L, MgCl_2_ 1 mmol/L, and CaCl_2_ 1.8 mmol/L. For pH 6.5, the buffer solution Pipes 5 mmol/L was used; Hepes 5 mmol/L was used to obtain a pH 7.6 and pH 8.5. The final pH values were adjusted with HCl or NaOH. The substrates tested were Gly-Gln, Ala-Ala, and Gly-Gly-Gly (Sigma-Aldrich). Every oligopeptide was added at the indicated concentrations (from 0.1 to 30 mmol/L) in the NaCl or TMA buffer solutions with appropriate pH.

### RNA extraction

RNA was extracted from adult tissues, embryos, and larvae by using the RNeasy® Plus mini kit (Qiagen) protocol, according to the manufacturer’s instructions, and implemented with the on-column PureLink DNase (Qiagen) treatment to eliminate possible genomic DNA contamination. Briefly, after removal of RNALater excess, tissues were lysed in the kit lysis buffer, until complete homogenization. At the end of the extraction protocol, RNA aliquots were stored at − 80 °C until use. RNA concentrations were calculated by spectrophotometry, and the *λ*_260_/*λ*_280_ ratios were calculated to evaluate possible protein contamination. The RNA was evaluated, qualitatively and quantitatively, in an agarose gel.

### Reverse transcription, RT-PCR and real-time PCR (qPCR)

For each total RNA extraction, two reverse transcriptions were performed on 500 ng RNA each, using the Bio-Rad iScriptTM Select cDNA Synthesis kit (Bio-Rad, Segrate, MI, Italy) and random primers according to the manufacturer’s instructions.

RT-PCR amplification assays were performed using Platinum® Taq DNA Polymerase (Thermo Fisher Scientific) according to the manufacturer’s protocol [10× PCR Buffer Minus Mg 5 μl; 10 mmol/L dNTP mixture 1 μl; 50 mmol/L MgCl_2_ 1.5 μl; Primer mix (10 μM each) 1 μl; Template cDNA ≥ 1 μl (as required); Platinum® Taq DNA Polymerase 0.2 μl; in a final volume of 50 μl]. A CFX96 Touch™ Real-Time PCR device (Bio-Rad) was used.

qPCR was performed using the IQ SYBR GREEN SUPERMIX protocol (Bio-Rad) on a CFX96 Touch™ Real-Time PCR device (Bio-Rad). Primer efficiencies in qPCR protocols for the expression of *pept1b* (*slc15a1b*), *pept1a* (*slc15a1a*), and the housekeeping gene *28S* were calculated according to the efficiency parameters proposed by [83]. Briefly, tenfold serial dilutions (1:1, 1:10, 1:100) of cDNA template were used in the presence of primers for the gene of interest and the *28S* rRNA. Threshold cycle (CT) output values (*y*-axis) were plotted vs. log of cDNA dilution (*x*-axis) to determine the slope of the line. qPCR efficiencies were then calculated by the equation *m* = −(1/log*E*), where *m* is the slope of the line and *E* is the efficiency. In the qPCR analysis, mRNA relative quantification was calculated analyzing the output CT values by the comparative CT method (also referred to as the 2^-ΔCT^ or 2^-ΔΔCT^ method [83, 84]); the qPCR data are shown as 2^-ΔCT^ values, which are taken as proportional to the amount of the target mRNA. ΔCT values (ΔCT = target gene CT − housekeeping gene CT) were obtained from two different rounds of qPCR (starting from two different retro-transcribed cDNA templates, each consisting of *n* = 3 biological replicates) for both the target and the *28S* internal control. According to [83], statistical analyses (see paragraph below) were performed after the 2^-ΔCT^ transformation.

Sequences and details on the specific primers used for PCR assays are reported in Additional file 1: Table S1.

### Statistical analysis

For functional analysis, descriptive statistic and logistic fit were applied; numbers of samples and of batch were reported in each figure. The analysis of the statistical significance between transport-associated currents under different experimental conditions was done using one-way ANOVA followed by Bonferroni’s post hoc multiple comparison test (differences were considered significant with at least *P* < 0.05). For embryos/larval stages, mRNA distribution analysis of the statistical significance among sample mRNA levels was done using one-way ANOVA followed by Tukey’s post hoc multiple comparison test (differences were considered significant with at least *P* < 0.05). All statistical analyses were conducted in R 3.5.1 [85].

## Supplementary information

**Additional file 1: Table S1.** List of the specific primers used for cloning and qPCR analysis. Sequence accession numbers, primer sequences and amplicon sizes are shown. **Figure S1.** Nucleotide and predicted amino acid sequence of zebrafish *pept1a* (*slc15a1a*) obtained using ORFfinder (https://www.ncbi.nlm.nih.gov/orffinder/). Numbers on the left refer to the nucleotide (upper row) and amino acid (lower row) positions. Nucleotides are numbered, starting from the first ATG initiation codon. * indicates the stop codon. The specific primers used for cloning and PCR analyses (Additional file 1: Table S1) are indicated in red and green, respectively. In the amino acid sequence, putative transmembrane domains, obtained using the TMHMM v. 2.0 program as implemented in SMART, are indicated and named I to XII. Potential extracellular N-glycosylation sites (white boxes), potential cAMP/cGMP-dependent protein kinase phosphorylation sites at the cytoplasmic surface (light gray boxes) and potential protein kinase C phosphorylation sites at the cytoplasmic surface (dark gray boxes) were obtained using the ScanProsite tool. **Figure S2.** Current-voltage relationships of transport-associated currents in zebrafish PepT1a, in the presence of 3 mmol/L Gly-Gln in sodium (NaCl) saline buffer (black square) and tetramethylammonium (TMACl) saline buffer (empty circle) at pH 7.6 (see **Methods** for details). Values are means ± SEM from 5 oocytes from one batch each group. The transport-associated current values reported were obtained by subtracting the current recorded in the absence of the substrate to that recorded in its presence. **Figure S3.** Expression analysis by RT-PCR on *pept1a* (*slc15a1a*) mRNA in different sections of adult zebrafish intestine. **a** RT-PCR assay on cDNA templates from total RNA extracted from whole gut (gut), intestinal bulb (I. bulb), mid intestine (mid) and posterior intestine (posterior); a PCR product of ~ 350 bp related to *pept1a* (*slc15a1a*) mRNA is present in all intestinal samples; L: 1 Kb Plus DNA ladder (Thermo Fisher Scientific). **b** A graphic representation of the adult zebrafish gut anatomy with its major adjacent tracts.

## Data Availability

Zebrafish *pept1a* (*slc15a1a*) nucleotide sequence has been submitted to GenBank (https://www.ncbi.nlm.nih.gov/nuccore/) and is available with the following accession number: GenBank Acc. No. [to be assigned]; GenBank Submission No. 2285160, via BankIt; release date June 21, 2020.

## Abbreviations

CDS: Coding sequence
Chr: Chromosome
GDV: Genome Data Viewer
*I*/*V*: Current/voltage
*I*_max_: Maximal transport current
*K*_0.5_: Apparent substrate affinity (i.e., apparent concentration of peptide that yields one-half of *I*_max_)
MS222: Tricaine methanesulfonate
PepT1: Peptide transporter 1 protein
*pept1a*: Peptide transporter 1a gene
PepT1a: PEPTIDE transporter 1a protein
*pept1b* or *pept1*: Peptide transporter 1b gene
PepT1b: Peptide transporter 1b protein
PepT2: Peptide transporter 2 protein
qPCR: Quantitative real-time PCR
Slc15a1 or SLC15A1: Solute carrier family 15 member 1 protein
*slc15a1a*: Solute carrier family 15 member 1a gene
Slc15a1a: Solute carrier family 15 member 1a protein
*slc15a1b*: Solute carrier family 15 member 1b gene
Slc15a1b: Solute carrier family 15 member 1b protein
Slc15a2: Solute carrier family 15 member 2 protein
TEVC: Two-electrode voltage clamp
UTR: Untranslated regions
WGD: Whole genome duplication
