# Correction to: The peptide transporter 1a of the zebrafish *Danio rerio*, an emerging model in nutrigenomics and nutrition research: molecular characterization, functional properties, and expression analysis

**DOI:** 10.1186/s12263-020-0661-7

**Published:** 2020-02-07

**Authors:** Francesca Vacca, Amilcare Barca, Ana S. Gomes, Aurora Mazzei, Barbara Piccinni, Raffaella Cinquetti, Gianmarco Del Vecchio, Alessandro Romano, Ivar Rønnestad, Elena Bossi, Tiziano Verri

**Affiliations:** 1 Department of Biotechnology and Life Sciences, University of Insubria, via J.H. Dunant 3, 21100 Varese, Italy; 2 Department of Biological and Environmental Sciences and Technologies, University of Salento, via Provinciale Lecce-Monteroni, I-73100 Lecce, Italy; 3 Department of Biological Sciences, University of Bergen, P.O. Box 7803, NO-5020 Bergen, Norway; 4 Present address: Physiopathology of Reproduction and IVF Unit, Nardò Hospital, Nardò Health and Social Care District, Lecce Local Health Agency, I-73048 Nardò, Lecce, Italy; 5 Division of Neuroscience, Institute of Experimental Neurology, IRCCS San Raffaele Scientific Institute, I-20132 Milan, Italy

**Correction to: Genes Nutr (2019) 14:33**

**https://doi.org/10.1186/s12263-019-0657-3**

Following publication of the original article [1], the authors flagged that the ‘Availability of data and materials’ declaration is incomplete.

Namely, it does not report the official accession number for the zebrafish PepT1a nucleotide sequence.

With concern to this, please see the (correct) ‘Availability of data and materials’ declaration here:

“Zebrafish *pept1a* (*slc15a1a*) nucleotide sequence has been submitted to GenBank (https://www.ncbi.nlm.nih.gov/nuccore/) and is available with the following accession number: GenBank Acc. No. MN723161”

The authors apologize for any inconvenience caused.
