# Supplementary material for: The peptide transporter 1a of the zebrafish Danio rerio, an emerging model in nutrigenomics and nutrition research: molecular characterization, functional properties, and expression analysis
Source: Genes Nutr. 2019 Dec 19;14:33. doi: 10.1186/s12263-019-0657-3 (PMC6923934; doi:10.1186/s12263-019-0657-3)
Supplement: Supplementary file 1 — Additional file 1: Table S1. List of the specific primers used for cloning and qPCR analysis. Sequence accession numbers, primer sequences and amplicon sizes are shown. Figure S1. Nucleotide and predicted amino acid sequence of zebrafish pept1a (slc15a1a) obtained using ORFfinder (https://www.ncbi.nlm.nih.gov/orffinder/). Numbers on the left refer to the nucleotide (upper row) and amino acid (lower row) positions. Nucleotides are numbered, starting from the first ATG initiation codon. * indicates the stop codon. The specific primers used for cloning and PCR analyses (Additional file 1: Table S1) are indicated in red and green, respectively. In the amino acid sequence, putative transmembrane domains, obtained using the TMHMM v. 2.0 program as implemented in SMART, are indicated and named I to XII. Potential extracellular N-glycosylation sites (white boxes), potential cAMP/cGMP-dependent protein kinase phosphorylation sites at the cytoplasmic surface (light gray boxes) and potential protein kinase C phosphorylation sites at the cytoplasmic surface (dark gray boxes) were obtained using the ScanProsite tool. Figure S2. Current-voltage relationships of transport-associated currents in zebrafish PepT1a, in the presence of 3 mmol/L Gly-Gln in sodium (NaCl) saline buffer (black square) and tetramethylammonium (TMACl) saline buffer (empty circle) at pH 7.6 (see Methods for details). Values are means ± SEM from 5 oocytes from one batch each group. The transport-associated current values reported were obtained by subtracting the current recorded in the absence of the substrate to that recorded in its presence. Figure S3. Expression analysis by RT-PCR on pept1a (slc15a1a) mRNA in different sections of adult zebrafish intestine. a RT-PCR assay on cDNA templates from total RNA extracted from whole gut (gut), intestinal bulb (I. bulb), mid intestine (mid) and posterior intestine (posterior); a PCR product of ~ 350 bp related to pept1a (slc15a1a) mRNA is present in all intestinal s [file 12263_2019_657_MOESM1_ESM.docx]

**Table S1** List of the specific primers used for cloning and qPCR analysis. Sequence accession numbers, primer sequences and amplicon sizes are shown.

| **Gene** | **GenBank Acc. No.** | **Sense primer 5’-3’ (Tm)** | **Antisense primer 5’-3’ (Tm)** | **Amplicon (bp)** |
| --- | --- | --- | --- | --- |
| **Cloning** |  |  |  |  |
| *pept1a* (*slc15a1a*) | NC_007120.7^&^ | CTTTCACACACACACTCTCT (52 °C) | AACAGACCCCTGTATCATCAT (53 °C) | 2478 |
| **RT-PCR** |  |  |  |  |
| *actb* | NM_131031.2 | CGTGACATCAAGGAGAAGCT (54 °C) | ATCCACATCTGCTGGAAGGT (55 °C) | 443 |
| *pept1a* (*slc15a1a*) | NC_007120.7^&^ | AGAACCGGCTGAGATGTAT (57 °C) | AAATACTGAGGAATCTGGAG (53 °C) | 351 |
| **qPCR** |  |  |  |  |
| *28S* | EF417169.1 | GGTCTAAGTCCTTCTGATGG (55 °C) | GGCTGCATTCCCAAACAAC (55 °C) | 112 |
| *pept1a* (*slc15a1a*) | NC_007120.7^&^ | AGAACCGGCTGAGATGTAT (57 °C) | GAAGGCTGAAGGCTGGACT (56 °C) | 132 |
| *pept1b (slc15a1b)* | NM_198064.1 | TGTGACCATCTCTGCTGGAG (56°C) | CCGCGTGCACATTATCAGAC (56°C) | 206 |

GC content, end stability, self/cross-dimer formation, and melting temperature of the oligonucleotides were analyzed by using the software AmplifX version 1.7.0 (<https://inp.univ-amu.fr/en/amplifx-manage-test-and-design-your-primers-for-pcr>). Amplification products from the primer pairs were sequenced and identified by alignment with the reference mRNAs. ^&^From GRCz11 (RefSeq Acc. No. GCF_000002035.6; GenBank Acc. No. GCA_000002035.4) Genome Assembly, Chr 9 (NC_007120.7): 1,136,369-1,163,151. Tm, melting temperature.

-44 ctttcacacacacactctctcacacacacgctcctgctgccaac

1 ATGCCAGACTCAAAGATGGACGAGACGAAGAAGAAGAAGAAAAAG

1 **M P D S K M D E T K K K K K KK**

46 ACGGCTGAGTGCTGTGGATATCCCATCAGCATCTTCTTCATTGTG

16 **T A E C C G Y P I S I F F I V**

<--------------------------------I-

91 GTCAATGAGTTCTGTGAGAGATTCTCCTATTATGGGATGCGCGCT

31 **V N E F C E R F S Y Y G M R A**

------------------------------->

136 GTGCTGGTGCTGTATTTCCGCTATTTTCTGCTGTGGGACGATGAT

46 **V L V L Y F R Y F L L W D D D**

181 CTGGCAACCTCCATCTACCATGCGTTCGTGGCGCTCTGCTACCTG

61 **L A T S I Y H A F V A L C Y L**

<--------------------------------II---------

226 ACGCCCATCCTGGGGGCCATCATCGCCGACTCCCGGCTCGGCAAG

76 **T P I L G A I I A D S R L G K**

---------------------->

271 TTCAAGACCATCATATACCTGTCTATAGTGTACGCAGTGGGGCAG

91 **F K T I I Y L S I V Y A V G Q**

<-------------------------------

316 GTGGTCATGGCCGTCAGCACTATTCATGACATCACTGACGCTAAC

106 **V V M A V S T I H D I T D A N**

III------------------------------->

361 AGAGACGGCACACCGGACAACTTCACCTTACACATTGCTCTCTCT

121 **R D G T P D N F T L H I A L S**

<-------------------------

406 ATGCTGGGTTTGGTCCTCATAGCTCTGGGCACCGGAGGAATTAAA

136 **M L G L V L I A L G T G G I K**

-------IV------------------------------->

451 CCGTGTGTTGCAGCGTTTGGTGGAGATCAGTTTCAGGAGCATCAG

151 **P C V A A F G G D Q F Q E H Q**

496 AGTCGGCAGCTCAACACTTTTTTCTCAGTGTTTTATTTGTGCATC

166 **S R Q L N T F F S V F Y L C I**

<-------------------------------

541 AACGCTGGAAGTCTGCTTTCCACACTCATCACGCCTGTGCTCAGA

181 **N A G S L L S T L I T P V L R**

V------------------------------>

586 GCTCAGGAGTGTGGCATCCACACGCAGCAGCAGTGCTATCCGCTT

196 **A Q E C G I H T Q Q Q C Y P L**

631 GCTTTCGGGGTCCCGGCAGCTCTCATGGTGGTGTCTCTGGTGGTG

211 **A F G V P A A L M V V S L V V**

<--------------------------------VI---------

676 TTTATTGCGGGCAGTGGCATGTACACCAAAACTGCTCCAGAGGGA

226 **F I A G S G M Y T K T A P E G**

---------------------->

721 AACATTATGGGCTCTGTGTGTAAATGCATATGGTTTGCTCTGAAT

241 **N I M G S V C K C I W F A L N**

766 AACCGTTTCAGACACCGAAGCGATATTTATCCAAAGAGGGAGCAC

256 **N R F R H R S D I Y P K R E H**

811 TGGATGGACTGGGCGGAGGAGAAATATGATAAACTCCTCATTGCG

271 **W M D W A E E K Y D K L L I A**

<----------

856 CAGATAAAGATGGTGCTGAAGGTGTTGTTCCTCTACATCCCCCTG

286 **Q I K M V L K V L F L Y I P L**

---------------------VII---------------------

901 CCCATGTTTTGGACCCTGTTTGACCAGAAGGGCTCCCGCTGGACT

301 **P M F W T L F D Q K G S R W T**

---------->

946 CTACAAGCCACCACCATGACCGGAGACTTTGGAGGGTTCGTCCTG

316 **L Q A T T M T G D F G G F V L**

991 CAGCCAGACCAGATGCAGACGGTGAACCCCATCCTCATCTTGACC

331 **Q P D Q M Q T V N P I L I L T**

<----------------------------

1036 CTGGTGCCCATCATGGACAGAATTGTTTTCCCTCTCATAAAAAAG

346 **L V P I M D R I V F P L I K K**

---VIII------------------------------>

1081 TGTGGCCTCAATTTCAGCCCTTTGAAGAGAATGACGGTCGGCATG

361 **C G L N F S P L K R M T V G M**

<-------------

1126 TTGTTCGCTGCCACAGCGTTTATTGCTGCTGCTCTGGTGCAGATG

376 **L F A A T A F I A A A L V Q M**

-----------IX------------------------>

1171 GAGGTTGATAAAACCTTGCCGAATTTCCCATCATCCTCTGAGAGC

391 **E V D K T L P N F P S S S E S**

1216 CAGCTGAAGGTGGTGAATATGCACAGCGAGTCTCTCATAGTGACT

406 **Q L K V V N M H S E S L I V T**

1261 GTGCCGTCCCAAGAGCCTCTACTGATCGGCTCATTTGAGAGCAGT

421 **V P S Q E P L L I G S F E S S**

1306 CCAGATTACATTACGTTTGGCCAGCAGGACATCAGGTTAGCCTTT

436 **P D Y I T F G Q Q D I R L A F**

1351 TACACAACTCCTGCGATCAATAAAGATTTGAGTTTAATCAAAGGC

451 **Y T T P A I N K D L S L I K G**

1396 AGCCGTCAGACCCTGATCATCCCCTCAGAACCGGCTGAGATGTAT

466 **S R Q T L I I P S E P A E M Y**

1441 CTGAAAGAAGACATCAAGTCTAAACCAAAGGAAGGGAAGAATGCT

481 **L K E D I K S K P K E G K N A**

1486 GTCAGGTTTGTAAACGGCTGGACTGCATATCTGAACATCACTAAC

496 **V R F V N G W T A Y L N I T NN**

1531 CTGGAGTCCAGCCTTCAGCCTTCAGAAACGTCAAACTACACGCTG

511 **L E S S L Q P S E T S N Y T LL**

1576 GTCTCTCAGGGCATGCGTAAGTTCACGCTAACCAATGGTATTCAG

526 **V S Q G M R K F T L T N G I Q**

1621 TCGTGTGAGTTTTCACGGAAGTTTGGCTTCGGTTCCTCCTACACT

541 **S C E F S R K F G F G S S Y T**

1666 TTCCTGATCCCCAGCGATCTGTTCTCCACTGATTGTGAGTCTATA

556 **F L I P S D L F S T D C E S I**

1711 AAGGAGATTGAAGACATGCAGCCCAACTCGGTGCACATGGCTCTC

571 **K E I E D M Q P N S V H M A L**

<-------

1756 CAGATTCCTCAGTATTTCCTCATCACTACGGGAGAGGTCATGTTC

586 **Q I P Q Y F L I T T G E V M F**

-------------------------X-------------------

1801 TCCGTCACCGGTCTGCAGTTCTCATACTCACAGGCTCCCAAAAAC

601 **S V T G L Q F S Y S Q A P K N**

------------->

1846 ATGAAGTCGGTGCTGCAGGCCGGCTGGCTGTGCACTAACGCAGTG

616 **M K S V L Q A G W L C T N A V**

<--------------------------------XI

1891 GGAAACATCATCGTGCTGATCGTGGCGGAGCTGGGGAAACTTCCC

631 **G N I I V L I V A E L G K L P**

------------------------------->

1936 AAACAGTGGGCAGAGTATGTGCTGTTTGCGTCGCTGCTAGTAGCT

646 **K Q W A E Y V L F A S L L V A**

<----------------------------

1981 GTTAGCATCATCTTCTCCATCATGGCGTATTTCTACACCTACATC

661 **V S I I F S I M A Y F Y T Y I**

--XII-------------------------------->

2026 GACCCAGCGGAGATTGAAGCCGAGATCCTGAAACAGCAAGAGACT

676 **D P A E I E A E I L K Q Q E T**

2071 GATCCAGACAAGAAGAAGAAGAAGGAGACTCTAGAAATGGAGGAA

691 **D P D K K K K K E T L E M E E**

2116 AAGGAGAACGAGCAGGAAATCAAACAAACCAAGATTTAAgacttg

706 **K E N E Q E I K Q T K I ***

2161 aagattgagttacgctcatgctaatatcttccctttacttccgta

2206 tttattaaagcaggtttgaaggactgtgagcagacgtgtgtgtgt

2251 ttatgattgatttgcatctgttgttgatacatttagatgattatt

2296 gatgagagccaattgagtcgacaatcatatgtagcattaaacatt

2341 taatgtgtaaaataaactttgttatggatatggattgtgatgtag

2386 atggactttagtgagtgtttttgtgtgtgttttgacttttttaag

2431 ctaataatcaatggactatgatgatacaggggtctgtt

**Fig. S1** Nucleotide and predicted amino acid sequence of zebrafish *pept1a* (*slc15a1a*) obtained using ORFfinder (<https://www.ncbi.nlm.nih.gov/orffinder/>). Numbers on the left refer to the nucleotide (upper row) and amino acid (lower row) positions. Nucleotides are numbered, starting from the first ATG initiation codon. * indicates the stop codon. The specific primers used for cloning and PCR analyses (**Table S1**) are indicated in red and green, respectively. In the amino acid sequence, putative transmembrane domains, obtained using the TMHMM v. 2.0 program as implemented in SMART, are indicated and named I to XII. Potential extracellular N-glycosylation sites (white boxes), potential cAMP/cGMP-dependent protein kinase phosphorylation sites at the cytoplasmic surface (light gray boxes) and potential protein kinase C phosphorylation sites at the cytoplasmic surface (dark gray boxes) were obtained using the ScanProsite tool.


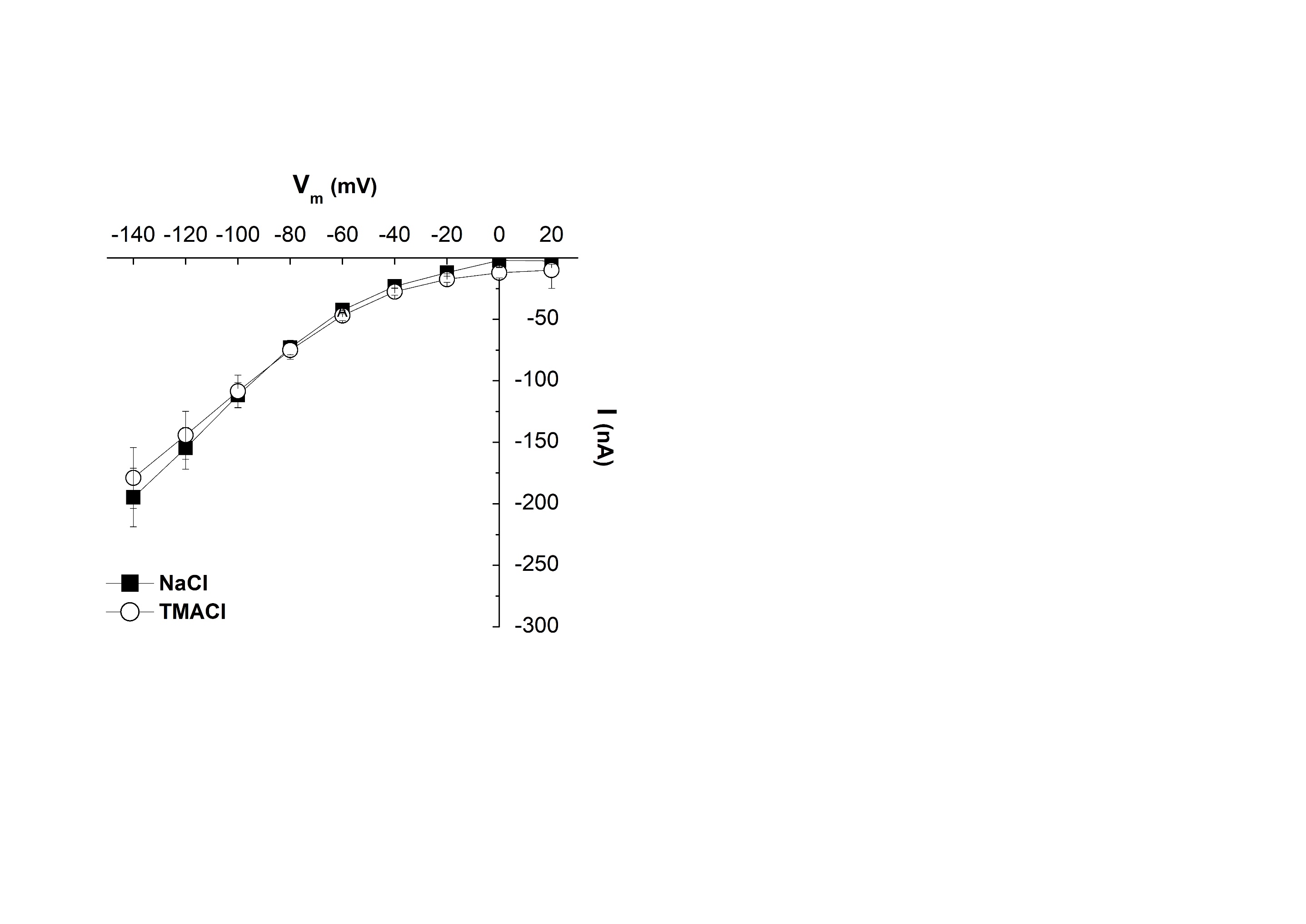


**Fig. S2** Current-voltage relationships of transport-associated currents in zebrafish PepT1a, in the presence of 3 mmol/L Gly-Gln in sodium (NaCl) saline buffer (black square) and tetramethylammonium (TMACl) saline buffer (empty circle) at pH 7.6 (see **Methods** for details). Values are means ± SEM from 5 oocytes from one batch each group. The transport-associated current values reported were obtained by subtracting the current recorded in the absence of the substrate to that recorded in its presence.


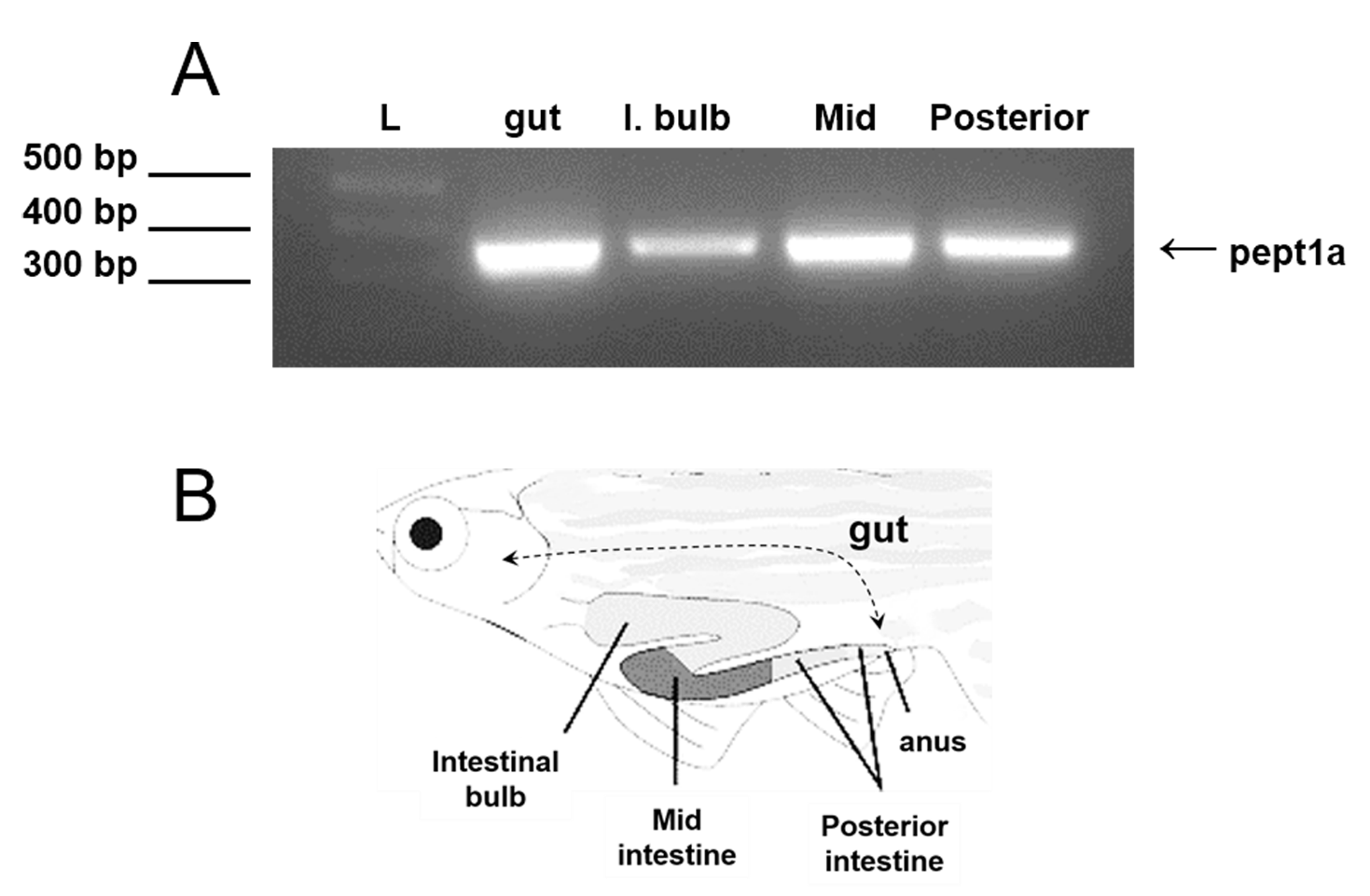


**Fig. S3** Expression analysis by RT-PCR on *pept1a* (*slc15a1a*) mRNA in different sections of adult zebrafish intestine. **a** RT-PCR assay on cDNA templates from total RNA extracted from whole gut (gut), intestinal bulb (I. bulb), mid intestine (mid) and posterior intestine (posterior); a PCR product of ~350 bp related to *pept1a* (*slc15a1a*) mRNA is present in all intestinal samples; L: 1 Kb Plus DNA ladder (Thermo Fisher Scientific). **b** A graphic representation of the adult zebrafish gut anatomy with its major adjacent tracts.
